# Dissecting microbial communities and resistomes for interconnected humans, soil, and livestock

**DOI:** 10.1038/s41396-022-01315-7

**Published:** 2022-09-23

**Authors:** Alexandre Maciel-Guerra, Michelle Baker, Yue Hu, Wei Wang, Xibin Zhang, Jia Rong, Yimin Zhang, Jing Zhang, Jasmeet Kaler, David Renney, Matthew Loose, Richard D. Emes, Longhai Liu, Junshi Chen, Zixin Peng, Fengqin Li, Tania Dottorini

**Affiliations:** 1 School of Veterinary Medicine and Science, University of Nottingham, College Road, Sutton Bonington, Leicestershire, LE12 5RD UK; 2 NHC Key Laboratory of Food Safety Risk Assessment, China National Center for Food Safety Risk Assessment, Beijing, 100021 People’s Republic of China; 3 New Hope Liuhe Co., Ltd., Laboratory of Feed and Livestock and Poultry Products Quality & Safety Control, Ministry of Agriculture, Beijing 100102 and Weifang Heshengyuan Food Co. Ltd., Weifang, 262167 People’s Republic of China; 4 College of Food Science and Engineering, Shandong Agricultural University, Tai’an, Shandong 271018 People’s Republic of China; 5 Nimrod Veterinary Products Limited, 2, Wychwood Court, Cotswold Business Village, Moreton-in-Marsh, GL56 0JQ UK; 6 DeepSeq, School of Life Sciences, Queens Medical Centre, University of Nottingham, Nottingham, NG7 2UH UK

**Keywords:** Metagenomics, Microbiome, Bacterial genetics, Bacterial infection, Infectious-disease diagnostics

## Abstract

A debate is currently ongoing as to whether intensive livestock farms may constitute reservoirs of clinically relevant antimicrobial resistance (AMR), thus posing a threat to surrounding communities. Here, combining shotgun metagenome sequencing, machine learning (ML), and culture-based methods, we focused on a poultry farm and connected slaughterhouse in China, investigating the gut microbiome of livestock, workers and their households, and microbial communities in carcasses and soil. For both the microbiome and resistomes in this study, differences are observed across environments and hosts. However, at a finer scale, several similar clinically relevant antimicrobial resistance genes (ARGs) and similar associated mobile genetic elements were found in both human and broiler chicken samples. Next, we focused on *Escherichia coli*, an important indicator for the surveillance of AMR on the farm. Strains of *E. coli* were found intermixed between humans and chickens. We observed that several ARGs present in the chicken faecal resistome showed correlation to resistance/susceptibility profiles of *E. coli* isolates cultured from the same samples. Finally, by using environmental sensing these ARGs were found to be correlated to variations in environmental temperature and humidity. Our results show the importance of adopting a multi-domain and multi-scale approach when studying microbial communities and AMR in complex, interconnected environments.

## Introduction

Antimicrobial resistance (AMR) is a major global concern and historically AMR surveillance has focused on clinical settings and high-income countries (HICs) [1]. However, resistant bacteria can circulate largely undetected outside of clinical settings, in healthy humans, livestock, and environmental settings, particularly in many low- and middle-income countries (LMICs) [1]. Compared to HICs, food production settings located in LMICs may be at higher risk, due to increased contact between livestock and humans [1, 2] and higher usage of antibiotics for metaphylaxis and growth promotion [3, 4]. Poultry represents a major source of animal protein for human consumption in the form of eggs and meat [5, 6]. The production of chicken meat has increased by over 1500% in the past 60 years [6, 7]. Growth in the demand for animal protein, due to an increasing human population has driven some countries, particularly LMICs, to shift towards highly intensive livestock production systems [8], where antimicrobials are routinely used to keep animals healthy and maintain productivity [3, 9, 10]. This is particularly true of China, where our study was conducted, a country that has seen an expansion of industrial livestock production over the past three decades [11]. China uses the largest proportion of antibiotics in animal production worldwide (23%) and use is projected to increase to 30% by 2030 [3].

Antibiotic usage, even at low levels, has been shown to alter and expand the gut resistome in livestock [12–14]. The gut resistome comprises the antibiotic resistance genes (ARGs) present in all the microorganisms lining the gut, including bacteria, viruses, archaea, and eukaryotes [15]. Whilst the gut microbiota is largely commensal and important for health, several studies have found that the microbiota can act as a reservoir for ARGs [16, 17], which in other studies have been found to be transferred between livestock and humans [18]. Several studies have found both direct (in food production) [19–25] and indirect (through food consumption) [26–28] evidence of similar antibiotic-resistant bacteria (ARBs) and ARGs between humans and animals/meat. However, the role of farm animals in the emergence and dissemination of ARBs and their resistance determinants to humans is poorly understood and controversial, with some studies comparing samples related on a regional level showing distinct human and animal lineages [29, 30]. Although food production settings are emphasized as high-risk transmission points [1], only a few studies to date have considered the AMR present in livestock, the humans in direct contact with them (i.e. farm/slaughterhouse workers), and their environment [19–22, 24, 25]. In addition, most of these investigations have been conducted in HICs [19–22] and there are few studies in LMICs, where risks are likely to be higher [1]. The greater human-animal contact and inadequate biosecurity, typical of LMICs, are likely to lead to increased risk of transmission among livestock, humans, and the environment, with different patterns of dissemination compared to HICs or hospital settings. Hence, similarities/differences across species may be missed by conventional surveillance approaches. It is therefore critical to set out studies and improved methods optimized to scenarios occurring in LMICs settings, to prospectively compare ARBs and ARGs from animals with those from humans. This will help to gather more evidence to understand if, where, and how AMR can reach humans [31, 32].

Culture-based methods using whole genome sequencing combined with antibiotic susceptibility testing (AST) and machine learning (ML) have proved to be a powerful prediction tool for genomic features associated with AMR [24, 33–37]. However, these approaches are limited to only those bacteria that are easily culturable, and in practice are focused on a few selected species [38]. Additionally, inconsistencies reported between whole genome sequence (WGS) based prediction using ML approaches and experimentally determined resistance/susceptibility profiles limit the applicability at present [39]. Importantly though, when studying complex resistomes that may be interconnected between hosts, investigating only a single or handful of species may not give the whole picture [40]. Further investigations in livestock, of antibiotic resistomes, encompassing all types of ARGs (acquired and intrinsic resistance genes) and mechanisms of overcoming taxonomic barriers within microbial communities, on livestock are needed [40]. However, these more comprehensive approaches could reveal important insights into ARG gene flow that cannot be observed using WGS methods, especially in these settings. For example, the movement of mobile elements carrying ARGs has been observed to be more influential on ARG flow than bacterial lineage in LMIC farms [41]. Hence, given the dynamic nature of the resistome [42], studying the core and mobile resistome, can provide an insight into which ARGs pose the greatest risks to humans [40]. Hu and colleagues [43] recently highlighted the extent of ARGs found in the vicinity of mobile genetic elements (MGEs) and showed that these mobile ARGs were most frequently exchanged between humans and livestock animals compared to other environments (aquatic and terrestrial), in a study based in China.

The gut resistome is complex and ever-changing. Tools that could help understand how such variability (e.g. type and abundance of ARGs [44]) relates to the resistance phenotypes of the commensal bacteria community and of pathogens inhabiting the gut, may help identify the ARGs that pose greater threat to public health. Analogously, by investigating ARGs that are correlated to resistance phenotypes of indicator bacteria, we can start to identify risk factors for hotspots of resistance beyond those directly linked to the ARGs present in the indicator pathogens. Exploring these research gaps may further help us to elucidate the complex relationship between the phenotypic presentation of AMR and the reservoir of ARGs within the resistome where the pathogen resides. An approach is to combine culture-dependent and culture-independent methods to observe correlations between phenotypic resistance and resistome [1]. ML has the capacity to efficiently analyse datasets with complex variables [45], making it ideally suited as a tool for studying AMR in this way.

Recently there have been studies combining both metagenomic and culture-based analyses [25, 46, 47] but none of these have correlated resistance/susceptibility phenotypes in cultured isolates with metagenomic data. Two recent studies used metagenomic samples to predict AMR and virulence determinants (by comparison of known genes in public databases) of clinical infections; however, typically these samples were mono-or polymicrobial with at most two species [48, 49]. Bacterial species such as *Escherichia coli* are already well established as important indicators of AMR in the wider microbiome context [50, 51]. Linking metagenomic data to the bacterial AMR phenotypes of important indicator species taken from the same samples may allow the species, genes, and MGEs correlated to AMR to be predicted. However, to date, there are no ML studies making use of metagenomic data in this way and we attempt to address this gap in the field.

Additionally, when linking phenotypic changes to the resistome, it is important to consider external factors, e.g. temperature or humidity, that may be involved as well. In physiological conditions the gut microbiome is stable, but when perturbative events occur (e.g., dietary changes, infections, stress, antibiotic administration, environmental changes) the population of the microbiome changes. These changes may involve new resistant bacteria becoming permanent residents or transferring resistance to the commensals [42, 52–54]. Whilst mesocosm studies would be required to fully elucidate factors influencing the resistome, limited information about specific factors that correlate with resistome changes is available and systemic approaches should integrate such data for a deeper analysis.

We hypothesize that: (i) in intensive food production environments located within LMICs, similarities and differences of resistomes and bacterial taxa distributions may be found in workers and animals, needing multi-scale analysis to be fully unravelled; (ii) correlations exist between resistance phenotypes of individual commensal and pathogenic bacteria and the types of ARGs in the resistome in which they reside; (iii) given the numerous environmental factors influencing gut modifications, AMR-correlated resistome is associated to various external factors (i.e. temperature and humidity); and (iv) an ML-powered approach that integrates culture-based AST, culture-independent methods (metagenomic data), and environmental sensing data, can be used to identify unknown associations between AMR phenotypes of cultured isolates, metagenome data from the same sample and external factors. To test these hypotheses we set three objectives: (i) perform a longitudinal study in a farm and connected slaughterhouse in China, to retrieve gut microbiome and resistome of livestock, workers, households, carcasses and soil, and unravel similarities and differences using multi-scale analysis; (ii) develop an ML-powered approach that integrates AST and metagenomics data to uncover correlations between ARGs in the chicken gut resistome and resistance phenotypes of individual gut microbiota members. To do this we have focused on *E. coli* in chickens, known to be an indicator species for AMR in livestock [50, 55], and for which we had the resistance/susceptibility profiles, to a panel of 28 antibiotics, obtained from *E. coli* isolates taken from the same samples as the chicken gut metagenome data; and (iii) assess whether the chicken gut resistome found to be correlated to the *E. coli* AMR phenotypes is itself associated to various external factors (i.e. temperature and humidity), which in intensive farming settings can be monitored over the lifetime of the livestock.

## Methods and materials

### Study farm and sample collection

All the broiler chicken samples were collected from a farm and connected abattoir in Shandong province, China. The farm implements self-breeding using a closed-end management model and contains on average 12,000 birds. The same-aged, batched broiler chickens (Ross 308 breed) were kept indoors in cages from birth, raised on the same feeds, and moved to the slaughterhouse on the day of slaughter. Samples were taken from two independent breeding cycles (spring cycle, timepoints denoted with a T, and summer cycle, timepoints denoted with an L). Between the breeding cycles, the feedlots were cleaned and dried. Temperature and relative humidity were manually recorded on the farm every 6 h using SMART SENSOR AS837. The readings were taken in three different positions within the barn and averaged. The barn’s temperature and humidity are subject to a heating/air conditioning system to maintain a relatively stable environment for growth, however, external conditions result in some uncontrolled variability.

For the broiler chicken faecal samples, 56 pooled samples were collected from week 3 (T1 and L1) and week 6 (T2 and L2). Among the collection, 36 samples were from the spring cycle and 20 were from the summer cycle. For each faeces sample, an approximately 10 g mixed fresh sample of broiler chicken faeces (2–3 broiler chicken faeces) was collected from the bottom of the broiler chicken cage using a sterilized spoon. Thirty-two broiler chicken carcass samples were collected at the slaughterhouse, during the slaughtering process (week 6 + 1 day, T3 and L3) using sponge swab (SS100NB, Hygiena International, Watford, UK) to swab the surface of the carcass. Twenty samples were from the spring cycle and 12 from the summer cycle. A total of 12 soil samples were collected from the barn exterior. For each of the soil samples, about 10 g of soil was collected at depth of 1–3 cm at 5 m from the broiler chicken barn away from human use. The 37 human stool samples consisted of 24 farm work samples (collected from 9 different full-time individual workers, including 1 vet), 8 abattoir full-time workers, and 5 household samples from their healthy relatives. Farmworkers had worked on the farm for between 1 and 10 years (mean 4.1 years) whilst the vet had worked on the farm for 11 years. The abattoir workers worked in the abattoir for between 6 and 19 years (mean 12.3 years), Supplementary Table 1. Specifically, for three farmworkers, samples were collected in the spring only at two timepoints (T1 and T2), for two farmworkers, samples were collected 4 times across spring and summer (T1, T2, L1, and L2), and for the remaining three farmworkers and vet, samples were collected either two or three times across both spring and summer (see Supplementary Table 1). Abattoir workers and their households were only sampled once. Sterilized sampling spoons were used to collect 8 g of each human stool sample. All samples were collected using aseptic techniques, and then stored in secure containers at 4 °C during transportation to the laboratory and extracted within 24 h.

### DNA library construction and sequencing

Samples were randomized for DNA extraction and sequencing to reduce batch effects. DNA extraction for soil and faeces samples was performed using a Magnetic Bead Genomic DNA Extraction Kit (DOP336-T3, TIANGEN Biotech (Beijing) Co. Ltd) and for carcass samples using the CTAB (cetyl trimethylammonium bromide) method [56]. Samples with tested DNA contents above 1 µg were used to construct the DNA library. The DNA concentration was measured using Qubit dsDNA Assay Kit in Qubit 2.0 Flurometer (LifeTechnologies, CA, USA) and the integrity was measured using 1% agarose gel electrophoresis. A total amount of 1 μg DNA per sample was used as input material for the DNA sample preparations. Sequencing libraries were generated using NEBNext Ultra DNA Library Prep Kit for Illumina (NEB, USA). The DNA sample was fragmented to 350 bp, then DNA fragments were end-polished, A-tailed, and ligated with the full-length adaptor for sequencing with further PCR. Finally, PCR products were purified (AMPureXPsystem) and libraries were analyzed for size distribution by Agilent2100 Bioanalyzer and quantified using real-time PCR. After cluster generation, the library preparations were sequenced on NovaSeq 6000 platform (Illumina) and 150 bp paired-end reads were produced.

### Antibiotic susceptibility testing of *E. coli* isolates

For each sample, where possible, *E. coli* strains were cultured as indicator organisms, tested against a panel of 28 antibiotics using broth microdilution and interpreted according to the criteria based on the Clinical & Laboratory Standards Institute (CLSI) interpretive criteria (CLSI 2009) (see Supplementary Methods for details).

### Bioinformatics analysis

The raw sequence reads were pre-processed and filtered using Readfq (V8, https://github.com/cjfields/readfq) to acquire high-quality data for subsequent analysis. Host DNA was mapped using Bowtie2 v2.3.4.1 and filtered out using SAMtools v1.9 [57] (reference genome accessions: GCF_000002315.6 (broiler chicken) and GCA_000001405.1 (human)). Microbiome assembly, binning, dereplication, and taxonomic assignment were performed using a similar pipeline to Glendinning et al. [58] (see Supplementary methods). Taxonomic classification and composition (relative species abundances) of the metagenome reads was performed in MetaPhlAn 3.0 [59] with Bowtie2 [60] using default settings, --bowtie2out –input_type fastq. Non-metric multidimensional scaling (NMDS) of the relative abundance at phylum level and separately species level was performed in R using the vegan [61] package with Bray-Curtis dissimilarity. The relative abundance was used only at a single level (i.e. phylum and separately species level) as the abundance at different levels is hierarchical and therefore inherently interdependent.

### Resistome analysis

Resistome analysis was performed on both full and rarefied data. Metagenome assemblies were compared using BLASTn [62] against the CARD database with a cut-off of 80% identity and 70% coverage [63]. Host removed reads were rarefied using the minimum sample depth (730158 reads in sample PDDTL3C2) using seqtk (https://github.com/lh3/seqtk), with the random seed fixed for each pair of reads. Rarefied reads were assembled using MEGAHIT [64], and rarefied assembled genomes were compared using BLASTn [62] against the CARD database with a cut-off of 80% identity and 70% coverage [63]. NMDS analysis was performed on the gene presence/absence data of the rarefied assemblies in R using the vegan [61] package using the Bray–Curtis dissimilarity.

### Analysis of common strains and MGEs across hosts

To look for the presence of similar mobile ARG content across different hosts, ARGs carried by both humans and broiler chickens were considered. ARGs were further selected if either: (a) they were considered as Rank I clinically important ARGs datasets according to Zhang et al. [44]; or (b) they were found in all three (humans, chickens, and soil) environments. For each selected ARG, the ARG-carrying contigs were then extracted (using the shell command grep) and filtered (>500 bp). Contigs were annotated using Prokka 1.14.6 and each encoded protein sequence was compared to the ISfinder database using BLASTn [62]. Annotated sequences were filtered, retaining only those sequences containing genes annotated as IS (i.e. transposases) based on a text search of the sequence annotations (TBL files). The distance between the end of the ARG gene and the start of the MGE (or vice versa) was calculated based on the annotated position (from Prokka), and ARG carrying contigs with a distance between ARG and MGE greater than 5 kb were discarded. Additionally, ABRicate (https://github.com/tseemann/abricate) was used to identify false positives, as a result of superimposed MGE and ARG sequences, by searching the IS sequences against the CARD database with 80% identity and 50% coverage. Any contigs with overlapping ARG and MGE sequences were discarded. The remaining contigs were considered as mobile ARGs as both an MGE and ARG were identified in the contig and the MGE was located within close vicinity (5 kb) of the ARG [25, 65–67]. The structure for the mobile ARG patterns (the MGE type, ARG type, distance, and sources) was summarized in Supplementary Table 8. For the mobile ARGs in common across hosts, the gene structure was visualized using gggenes in R.

For species present in > 75% of samples in all hosts, strain-level analysis was performed using StrainPhlAn [68]. The MetaPhlAn output was used as input to StrainPhlAn. Default parameters with ‘--phylophlan_mode accurate --mutation_rates’ were used. The resulting alignment was used as input to IQtreev2.0.3 [69] using model selection and 10000 ultrafast bootstrap replicates. In addition, *E. coli* MAGs from 12 human and broiler chicken metagenome samples were further analyzed, as well as the WGS from 76 human and chicken *E. coli* strains cultured from the same samples (30 human and 46 chicken) for which the metagenomic analysis was done [24]. For the *E. coli* MAGs and separately *E. coli* WGS, phylogenetic trees were reconstructed. Firstly, the MAGs and WGS were annotated with Prokka v1.14.5 [70] using default parameters with --addgenes --usegenus. Next, the annotated sequences were used as input to generate core genome alignments using Roary v3.13 [71] with default parameters. These alignments were then used to construct maximum likelihood phylogenetic trees using IQTreev2.0.3 [69] with model selection and 10000 Ultrafast bootstrap replicates. All the resulting trees were plotted in iToLv5 [72].

### Machine learning classification

Machine learning was used to identify the ARGs present in the faecal metagenomes that were associated with the antibiotic resistance/susceptibility profiles of the cultured *E. coli* isolates taken from the same samples. The analysis was performed on chicken faeces only. The human, soil, and carcass samples were neglected as there were too few samples to robustly test and train the ML models. ARG presence/absence was used as predictors (features) for the ML models. The resistant/susceptibility profiles for a panel of 28 antibiotics were the variables being predicted, with each antibiotic analyzed independently.

The supervised ML pipeline consists of three stages: (i) feature selection, (ii) classification, and (iii) post-processing analysis. For the feature selection stage, the Python package Scikit-learn [73] was used. We applied a synthetic minority over-sampling technique (SMOTE) [74] to overcome unbalanced classes in the variables being predicted (AMR resistance/susceptibility profiles) and the low number of samples in this work. SMOTE oversamples the minority class using the five nearest neighbours, increasing the overall number of samples with synthetically generated data. Features (predictors) were further reduced using a chi-squared test. All the features with a *p* value higher than 0.01 were removed, leaving only those most strongly associated with the variables being predicted (AMR resistance/susceptibility profiles). No multiple-comparison correction was used as we were looking to assess each feature in its own right [75]. The features selected for each antibiotic model were visualised in an undirected graph using the NetworkX [76] library in Python. Each node represented either the antibiotic or the feature (ARG). An edge connecting two nodes indicates that the feature is predictive of the antibiotic classification result. The nodes were positioned according to the Kamada-Kawai path-length cost function.

For the classification stage, a panel of ML methods consisting of 5 classifiers (logistic regression, linear support vector machine, radial basis function support vector machine, extra tree classifier, and random forest) and 2 meta-methods (AdaBoost and XGBoost) were implemented using the Python package Scikit-learn [73]. The AdaBoost (adaptative boosting) algorithm is a boosting algorithm that combines multiple weak classifiers to build a stronger classifier, in this case a decision tree is used as the classifier to be boosted. The XGBoost (gradient boosting) classifier builds an additive model by optimizing a binomial loss function; since we are analyzing a two-class problem, a single regression tree is induced and at each stage of the learning this tree is fitted on the negative gradient of the loss function. AMR resistance/susceptibility profiles of the *E. coli* isolates were used as variables being predicted for the classification. The ARGs (presence/absence) selected in the previous feature selection stage were used as predictors for the classification. Nested Cross-validation (NCV) [77] was employed to assess the performance and select the hyper-parameters of the proposed ML methods. In NCV, an outer loop split the data set into test and training sets. For each training set, a grid search (inner loop) was run, to find the best hyper-parameters. The inner loop of the NCV found the best hyper-parameters of each classifier using stratified 2-fold cross-validation and trained the model; the outer loop measured the receiver operating characteristics: area under the curve (AUC), accuracy, sensitivity, specificity, and precision of the test data set (unseen in the inner loop for the training) using 4-fold stratified cross-validation, to compare all the ML methods [78]. Thirty iterations of each classifier were carried out, and in each iteration, an NCV was employed. The final performance was evaluated with the following metrics: AUC, accuracy, sensitivity, specificity, and precision using the mean of 30 iterations of the NCV. To assess which method performed best the Friedman Statistical F-test with a Nemenyi *post-hoc* test was employed and showed the logistic regression to be the best classifier for these datasets (see Supplementary Material).

To assess if the ARGs associated with the *E. coli* AMR resistance/susceptibility profiles were impacted by changes in the environmental temperature and humidity, a second pipeline was created. This pipeline correlated the features selected in the first stage (feature selection) of the ML pipeline (i.e. ARGs from 56 broiler chicken faeces samples correlated via chi-squared to test the *E. coli* AMR profiles) with temperature/humidity using linear least-square regression analysis. The analysis was conducted only for the broiler chicken faeces samples as the chickens were confined to a monitored climate-controlled environment. The features were used to assess the correlation of each feature with mean temperature/humidity (mean of the seven days preceding the sample collection) measured over four different collection dates (T1, T2, L1, and L2). Each feature was used as an input for linear least-square regression analysis (using the Python package Scipy) with the dependent variable being either the temperature or the humidity. Features (ARGs) were significantly correlated with temperature or humidity if the slope of the regression line statistically differed from 0 (*p* value < 0.05 using a *t*-test).

### Analysis of antibiotic usage bias on the correlation of ARGs with temperature and humidity

The broiler chickens in the two production cycles received different antibiotics with spring broiler chickens receiving amphenicol antibiotics and summer broiler chickens receiving beta-lactam antibiotics. Additionally, both production cycles used aminoglycosides (Supplementary Table 12). To analyse if the differences in antibiotic treatment in each production cycle led to bias in the ARGs, the number of ARGs in each antibiotic class for each cycle was calculated and differences between the two production cycles were tested with a Mann–Whitney *U* test.

## Results

### Assembly and construction of microbial genomes

**Fig. 1:**
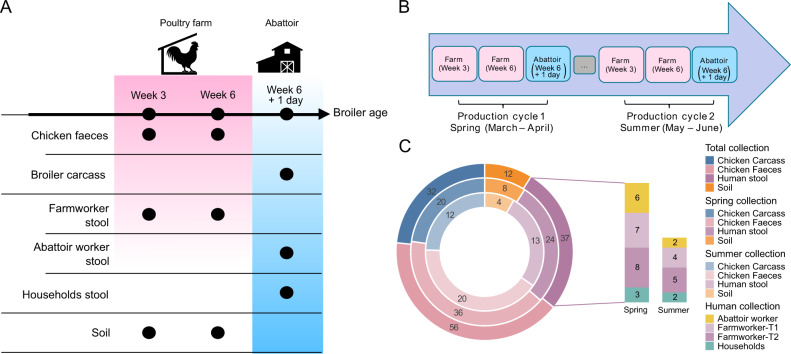
Overview of the study design and of the sampling collection through the broiler chicken production cycles. **A** Broiler chicken, carcass, human and soil samples were taken during different weeks (3 and 6) in the broiler chicken production cycle, and at 6 weeks + 1 day in the abattoir. The black dots highlight the collection timepoints and source location (farm or slaughterhouse) for each type of sample. **B** Two production cycles were collected, March to April 2019 (spring) and June to July 2019 (summer). All samples were named with reference to the collection timepoints: T1 (week 3, mid-growth broilers), T2 (week 6, fully grown broilers) and T3 end of life (week 6 + 1 day, slaughtered) for the first production cycle; whilst L1, L2, and L3 were used for the sampling during the second production cycle. **C** Distribution of the samples from the different sources across the total collection (outer ring), spring collection (middle ring), and summer collection (inner ring). Colour indicates sources while the number indicates the count of samples from that source. Human stool collections are further broken down by type and time.

We collected 137 samples from both farm and slaughterhouse, targeting workers (*n* = 32) and their households (*n* = 5), livestock (*n* = 56), carcasses (*n* = 32), and soil (*n* = 12) (Fig. 1A and Supplementary Table 1). Samples were taken over two independent production cycles, with some humans sampled across multiple timepoints and production cycles (Fig. 1B, C, Supplementary Table 1). Sequencing generated a median of 89 million (range 78M–189M) raw reads and 88 million (range 74M–188M) trimmed reads across all samples. After host removal from human and chicken samples, trimmed sequencing depth was reduced to a median of 81 million (range 1.4M–168M) reads. Comparing the two production cycles, whilst the raw reads and trimmed reads were statistically different (t-test *p* values < 0.001), the host removed reads used for further analysis did not differ (t-test *p* value 0.53). Comparing different source types (chicken faeces, human faeces, chicken carcasses, and soil) no statistical differences were observed for raw or trimmed reads (ANOVA *p* values > 0.05). However, host-removed reads differed significantly by source (ANOVA *p* value < 0.0001), and *post hoc* testing indicated that this was due to the chicken carcasses sequencing depth being significantly lower than other source types (Tukey test *p* values < 0.0001). Assembly quality of the samples varied by sample type with chicken carcass and soil samples having relatively poor assemblies, with median N50 values of 1001 and 833 respectively, whilst chicken faeces (median N50 = 2990) and human faeces (median N50 = 6293) samples were much better. Additionally, the percentage of reads mapped to assemblies was lower for soil (mean 41%) and carcasses (75%) compared to the chicken and human faeces samples (98%), highlighting the differences in assembly quality between the sample sources. Assembly quality, as assessed by the number of contigs, total contig length, GC content, N50 and read mapping rate, did not differ between the two production cycles (t-test *p* values > 0.05).

The dereplicated sets of metagenome-assembled genomes (MAGs) comprised 566 genomes from broiler chicken faeces and 16 from broiler chicken carcasses (Supplementary Table 2); 574 from human faeces (Supplementary Table 3); and 84 MAGs from soil (Supplementary Table 4). After MAG construction, an average of 38%, 71%, 31% and 44% of the assembly sequences of chicken faeces, chicken carcass, human faeces and soil samples, respectively, remained unbinned. The low number of MAGs from the chicken carcasses may reflect the low sequencing depth and poor assembly of these samples. The phylogeny of the chicken MAGs was reconstructed (Fig. S1A) and the novelty of the MAGs found in this study were compared to those present in public databases and a previous UK-based study [58] (Supplementary Material). Taking data from a previous study of 178 chicken faecal MAGs generated from 9 European countries [79], and mapping the reads back to our MAGs, we found that 75% of reads were mapped to our MAGs, demonstrating a high level of commonality (Figs. S2 and S3). Although the carcasses were collected after the disinfection process (chilling), bacterial MAGs from carcasses could still be identified (Fig. S1B). The phylogeny tree of the 574 human faecal MAGs was reconstructed (Fig. S1C). We performed pairwise comparisons between the chicken and human MAGs and found that 6 MAGs clustered at greater than 99% ANI, representing strain-level MAG clusters [58, 80, 81]. From soil samples, 84 MAGs were identified as bacteria (Fig. S1D).

### Abundance and diversity of the microbial community structure reveal a source and temporal pattern variation in the farm and slaughterhouse

**Fig. 2:**
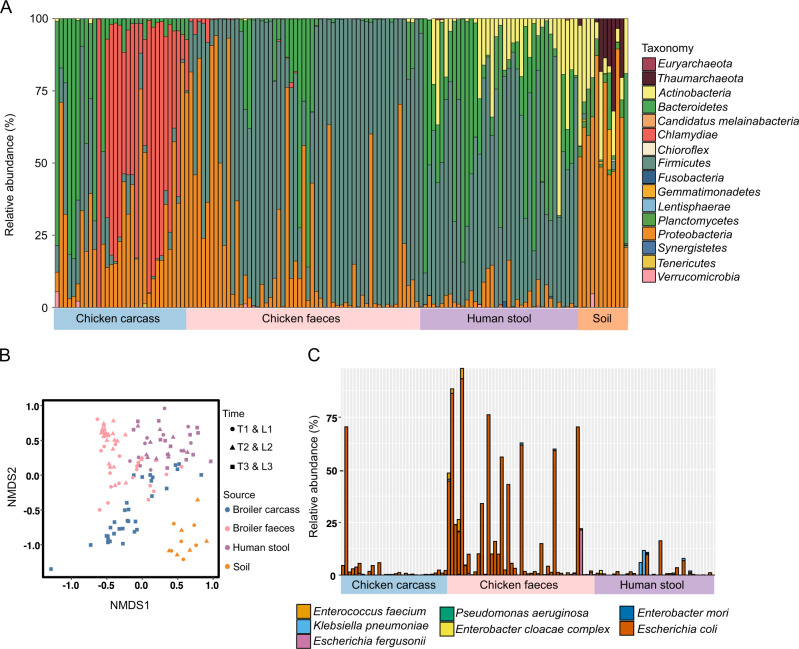
Composition of humans, soil, livestock, and carcasses microbiomes under different sources and time. **A** Taxonomic classification and composition of metagenomes from individual carcass, chicken faecal, human faecal, and soil samples. The relative abundances of taxa at the phylum level across the samples are compared. **B** NMDS analysis of the bacterial communities in broiler chicken carcasses (blue), broiler chicken faeces (pink), human faeces (purple), and soil (orange), based on Bray–Curtis dissimilarity at timepoints T1 and L1 (circles), T2 and L2 (triangle), T3 and L3 (squares). **C** Relative species abundance of the EKSCAPE pathogens present in different samples. These pathogens were not present in any soil samples.

After taxonomic profiling, 14 bacteria phyla were identified within the dataset of metagenomic samples (Fig. 2A and Supplementary Table 5). At the species level, we found 115 species in chicken faeces, 138 species in chicken carcasses, 390 species in human faeces, and 126 species in soil. Comparing human samples to chicken samples, 14% of species, 50% of bacterial families, and 45% of phyla were found in common between these two sources. When also considering species abundance, by using NMDS ordination for relative abundances of phyla, the bacterial communities from different sources were found to cluster separately (PERMANOVA *p* value < 0.001, pairwise adonis *p* values < 0.01), Fig. 2B. Temporal patterns in human, broiler chicken, soil, and carcass microbial community structures revealed a modest change at the three timepoints (Fig. 2B). At species level similar patterns were observed with species abundance clustering separately by source (Fig. S4) (PERMANOVA *p* value < 0.001). Chicken gut species composition had a statistically significant difference (PERMANOVA, *p* value <0.001) both within the same cycle (between samples taken at T1 compared to T2 and L1 compared to L2), as well as between cycles (between samples taken at T1 compared to L1 and T2 compared to L2) (Fig. S5). *Escherichia coli, Lactobacillus johnsonii*, and *Lactobacillus salivarius* were the most abundant species present over time in all the 56 broiler chicken samples (Supplementary Table 5). Opportunistic pathogens were found, including 4 ESKAPE pathogens [82] (*Enterococcus faecium, Klebsiella pneumoniae, Pseudomonas aeruginosa*, and *Enterobacter* species) and *E. coli*, in broiler chicken faeces, broiler chicken carcasses and human samples (Fig. 2C).

### Differentiated resistomes were found in gut bacteria of farm workers, slaughterhouse workers, chickens, soil, and broiler chicken carcasses

**Fig. 3:**
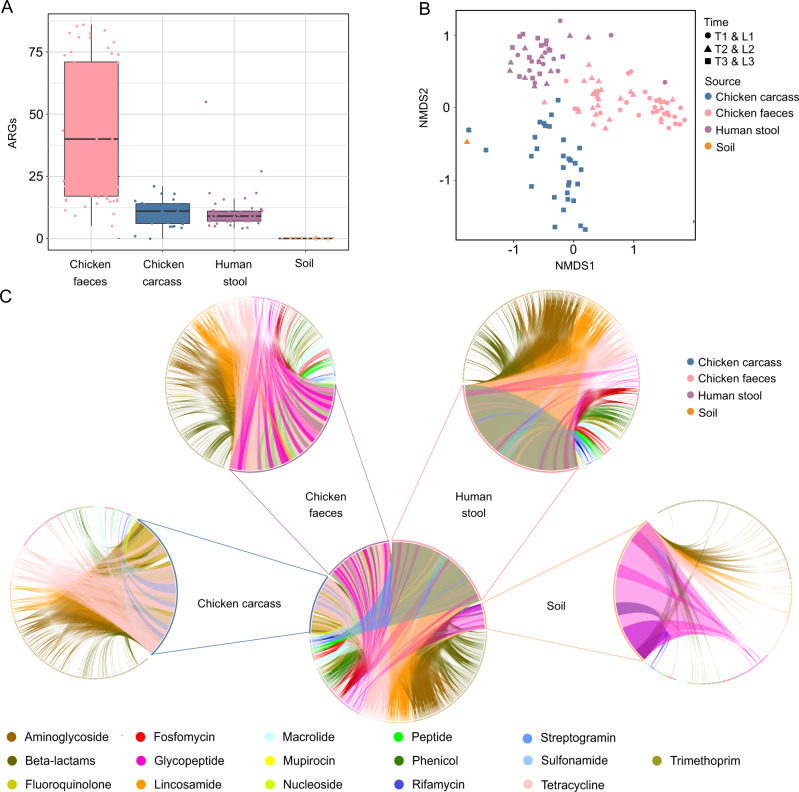
Occurrence of antibiotic resistomes in humans, soil, livestock, and carcasses under different sources and time. **A** The abundance of the ARGs in broiler chicken carcass, broiler chicken faeces, human faeces, and soil samples, data were rarefied to minimum read depth. **B** NMDS analysis of the resistomes in broiler chicken carcasses (blue), broiler chicken faeces (pink), human faeces (purple) and soil (orange), based on Bray-Curtis dissimilarity at timepoints T1 and L1 (circles), T2 and L2 (triangle) T3 and L3 (squares), data were rarefied to minimum read depth. **C** Circos plots of relative abundance and compositions of ARGs in broiler faeces, broiler carcass, human faeces, and soil samples.

In total 9171 ARGs were characterized and grouped into 360 ARG types (Supplementary Table 6), representing 0.02% of the combined gene sequences across all samples. After rarefying the data to correct for sequencing depth effects, the number of ARGs varied between sources (broiler chicken faeces, broiler chicken carcasses, human faeces, and soil), Kruskal–Wallis test *p* value < 0.0001 (Fig. 3A). Similarly, the diversity of ARGs varied between sources (PERMANOVA *p* value < 0.0001) (Fig. 3B). In *post-hoc* testing, the number of ARGs in broiler chicken faeces was found to be significantly different to all other sources (Dunn’s test with Bonferroni correction, *p* values < 0.0001). ARG number in human faeces was not significantly different from soil (Dunn’s test with Bonferroni correction, *p* value = 0.0732) or broiler chicken carcass (Dunn’s test with Bonferroni correction, *p* value = 0.650). Boiler chicken carcass and soil were significantly different from each other (Dunn’s test with Bonferroni correction, *p* value = 0.001). The diversity of ARGs in the rarefied data was significantly different across all sample sources using *post-hoc* pairwise adonis comparisons (*p* values, with Bonferroni correction < 0.01). Within the rarefied data, 71 of the 93 ARGs found in human samples (76%) were also found to be present in samples from chickens. All humans in the study were found to carry at least four ARGs that were also present in chicken samples (range = 4–55, mean = 9). The ARGs detected in broiler chicken faeces microbiota, broiler chicken carcass microbiota, and human faeces microbiota each related to the resistance to 13 classes of antibiotics, whereas the ARGs detected in soil microbiota related to 11 classes of antibiotics, with no fluoroquinolone or peptide ARGs detected, (Fig. 3C, Supplementary Table 7). Over their life cycle, the broiler chickens were treated with only aminoglycoside, amphenicol (spring only), and penicillin-based (summer only) antibiotics, hence resistance to the other classes of antibiotics may reflect indirect selection or a ubiquitous presence of these genes. We identified a wide diversity of acquired tetracycline resistance genes, aminoglycoside genes, and extended-spectrum beta-lactamase genes in human and broiler chicken faeces, and broiler chicken carcass samples. Six quinolone genes were found in both human and broiler chicken samples, the most prevalent of these being *QnrS1* found in 51 broiler chicken and 8 human samples.

As shown by the NMDS analysis, the ARG presence/absence profiles had significant variation between different sample sources (broiler chicken faeces, human faeces, broiler chicken carcasses, and soil). Compared to the microbial species abundances, the ARGs were more separated by source. Considering temporal variation, the broiler chicken faecal resistome showed minor intra-sample variation (Fig. 3B), with the samples taken at the two timepoints, week 3 (T1 and L1) and week 6 (T2 and L2), tightly clustered and intermixed. Analogously, the human gut resistome showed minor divergence between the samples taken at the three timepoints (farm and abattoir). The broiler chicken carcass resistome composition showed the highest intra-cluster divergence and inter-cluster separation exhibiting dissimilarities from workers and chickens resistomes (Fig. 3B).

### Evidence of similar clinically relevant mobile ARGs amongst the chicken and human hosts and similar *E. coli* strains amongst hosts

**Fig. 4:**
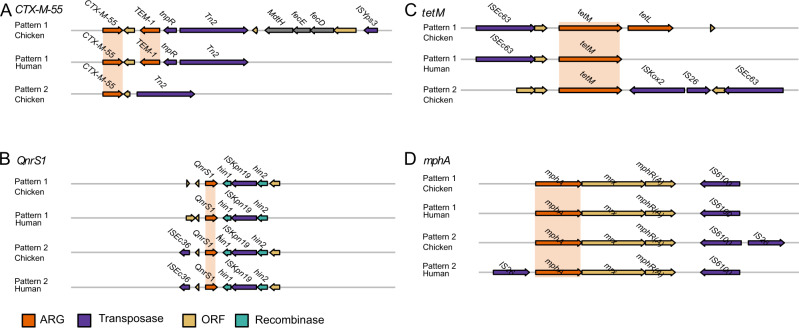
Genomic structure patterns of mobile ARG patterns present in both broiler chicken and human samples with identified ARG and mobile genetic elements. Four exemplar ARGs **A** *CTX-M-55*, **B** *QnrS1*, **C** *tetM* and **D** *mphA* are illustrated and aligned by the selected ARG for each pattern, shaded in light orange. Coding DNA sequences (CDSs) are coloured according to the function of their encoded protein, as follows: ARGs were coloured in orange, transposases were coloured in purple, recombinases were coloured in green and other open reading frames (ORF) were coloured in yellow.

We considered whether the same clinically relevant mobile ARGs were found in different hosts. We classified contigs with both ARGs and MGEs as mobile ARGs as previously done by Sun et al. [25]. A length restriction of 5 kb was then applied between the ARGs and MGEs to confine the analysis to ARGs located in the direct vicinity of MGEs. Both transposases and IS unit transposons were found close to ARGs. Of the 73 clinically relevant ARGs [44] considered (see Methods and Materials), 11 (approximately 15%) were found as mobile ARGs in both chicken and human samples (Supplementary Table 8). Additionally, of the 15 non-clinically relevant ARGs found in all three hosts (see Materials and methods), three were present as mobile ARGs found in both chickens and humans (Supplementary Table 8). In those cases where mobile ARGs were found in both humans and chickens, the humans were predominantly farmworkers (five). Additionally, one slaughterhouse worker and one household member carried mobile ARGs also found in chickens. No mobile ARGs found in either humans or chickens were also found in soil. The distance between the ARG and MGE was found to be between 0.01–3.46 kb with a mean distance of 0.97 kb. *CTX-M-55*, a beta-lactamase gene commonly found in poultry in China was found in two human samples and in one of these samples (human household) the gene and MGE aligned with broiler chicken samples (Fig. 4A). The clinically important *QnrS1* gene was widely present in chicken and human samples and had two different mobile ARG patterns: (i) the transposase *ISKpn19*, present in 41 broiler chicken samples and one human sample; and (ii) a pattern with the additional transposase *ISCc36*, present in two broiler chicken samples and one human (Fig. 4B). The most diverse ARG found as a mobile ARG was *bla*_*TEM-1*_, three mobile ARG patterns were found in both human and chicken hosts, and this gene was found in 12 different mobile ARG patterns altogether (Fig. 4A and Supplementary Table 8). The mobile *tetM* gene was found to be associated with two mobile ARG patterns (Fig. 4C). A mobile ARG containing the macrolide ARG *mphA* and the transposase *IS6100* was present in the highest number of humans with this ARG found in five human samples and 37 broiler chicken samples (Fig. 4D).

**Fig. 5:**
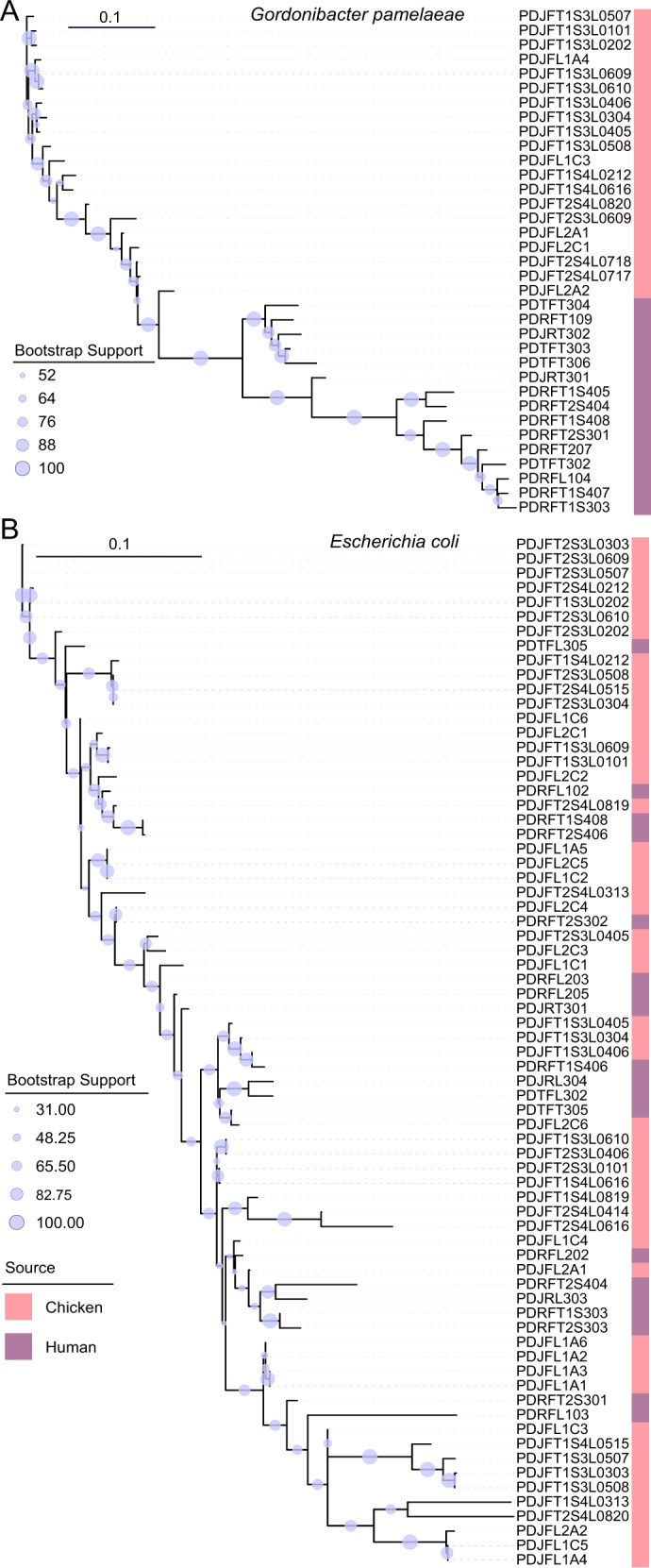
Strain level analysis with StrainPhlAn showing the relationships for human and broiler faeces metagenome. **A** Phylogenetic tree of *Gordonibacter pamelaeae* strains. **B** Phylogenetic tree of *E. coli* strains. Bootstrap support values are indicated by the size of the blue coloured circles.

To gain a deeper insight into the potential commonality of microbiota across hosts we looked at strain level population structure using StrainPhlAn. Only two species were present in a sufficient proportion of samples (>75%) that allowed the analysis of the strain-level structure: *Gordonibacter pamelaeae*, present in low abundance (range 0–1.4%) in 74 samples (30 human and 44 chicken faeces) and *E. coli*, present in much higher abundances (range 0–93.8%) in 88 samples (32 human and 56 chicken). The *G. pamelaeae* tree, Fig. 5A, showed conserved clusters of broiler chicken and human samples. However, the *E. coli* tree, Fig. 5B, showed mixing of clusters indicating a close phylogenetic relationship between strains in different hosts. Using a threshold of a normalized phylogenetic distance of 0.1 as the definition of strain identity, as used in other studies, we saw an overlapping of strains in human and chicken hosts with 506 human-chicken strain pairs. As the bootstrap support was poor for the *E. coli* tree, further analyses were conducted to corroborate the observed mixed clusters of human and broiler chicken strains. Firstly, a maximum likelihood tree of the 12 *E. coli* MAGs recovered from the metagenome samples again showed mixed clusters of human and broiler chicken *E. coli* strains. Though most nodes had bootstraps > 90%, nine nodes showed weak support with bootstraps <90%, Fig. S6. Secondly, using 76 WGS of cultured *E. coli* strains isolated from the same samples (46 chicken and 30 human) [24] for which the metagenomic analysis was done, the reconstructed phylogeny of these isolates again showed mixed clusters of human and broiler chicken strains with high bootstrap support (95% of nodes with support >90%), Fig. S7. Taken together, these analyses suggest that there are close phylogenetic relationships between some *E. coli* strains in different hosts.

### Supervised machine learning correlates resistance/susceptibility *E. coli* profiles to the chicken faeces resistome

As *E. coli*, a notable drug-resistant bacteria of great concern worldwide, was found to be abundant in both human and broiler chicken faeces and with similar strains found in the two hosts, we further investigated if there was also a correlation between the chicken gut resistome and the antimicrobial resistance/susceptibility phenotypes of *E. coli* isolates taken from the same samples as the metagenome data. We wanted to address the question of what genes within the resistome were correlated with the AMR (resistance/susceptibility) profiles we observed. We cultured and characterized 46 *E. coli* isolates from broiler chicken faeces (for which we also had metagenomic data) and evaluated their AMR profiles against a panel of 28 antimicrobials [24] via laboratory testing (Materials and methods). Human, soil, and broiler chicken carcass samples were not included in the analysis as there were too few isolates (with related metagenomic data) to robustly train and test the ML models in each category (soil n = 7, carcass *n* = 19, and human *n* = 30).

**Fig. 6:**
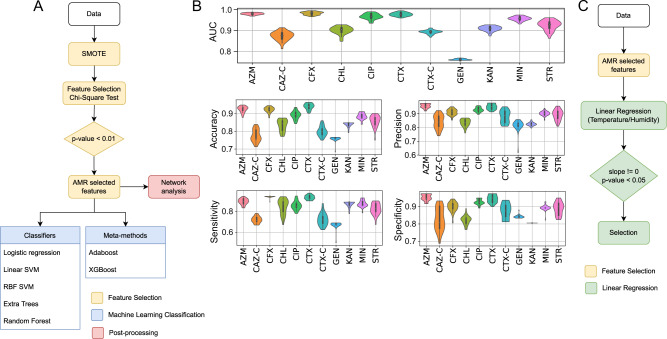
Workflow of the ML pipeline and prediction performance results correlating the broiler chicken resistome with the AMR resistance/susceptibility profiles on the farm, and regression analysis flow diagram to correlate the local temperature and humidity to the resistome. **A** Supervised ML pipeline used to search for correlations between ARGs (features) present in the broiler chicken faecal metagenomes and the antimicrobial resistance/susceptibility profiles of cultured *E. coli* isolates from the same sources. The pipeline consists of three stages, feature selection (shown in yellow), classification (shown in blue) and postprocessing analysis using an undirected graph network (shown in red). First, a synthetic minority over-sampling technique (SMOTE) was used to balance the data and a chi-square test was used as a feature selection method to select the features more correlated with the AMR phenotype. Next, for the classification stage, a panel of ML models consisting of 5 classifiers (logistic regression, linear support vector machine, radial basis function support vector machine, extra tree classifier and random forest) and 2 meta-methods (adaboost and xgboost) were used to predict the AMR phenotype based on the presence/absence of ARGs from the chicken broiler faecal metagenomes. **B** Prediction performance results of the classification, five performance indicators have been used to evaluate the ML models: AUC (area under the receiver characteristic operation curve), accuracy, sensitivity, specificity, and precision. These are generated from 30 iterations of the nested cross-validation results. The ML models were run for the following antibiotics: aztreonam (AZM), cefotaxime (CTX), cefotaxime/clavulanic acid (CTX-C), cefoxitin (CFX), ceftazidime/clavulanic acid (CAZ-C), chloramphenicol (CHL), ciprofloxacin (CIP), gentamicin (GEN), kanamycin (KAN), minocycline (MIN), streptomycin (STR). **C** Regression analysis pipeline to investigate whether the ARGs associated with AMR resistance/susceptibility profiles on the farm (those selected by the feature selection step, **A**, in the ML pipeline) were also correlated to the environmental temperature and humidity.

Using a supervised ML-based approach (Fig. 6A), we used ARG presence/absence data for each sample as predictors (features) to train and test prediction models to classify *E. coli* AMR (resistance/susceptibility) profiles of each antibiotic. To reduce the feature space, a rigorous feature selection process was applied (see Materials and methods). A minimum of six samples in the minority class were required for the classification. Therefore 15 antibiotics (AMP, AMS, TET, SXT, CFZ, IMI, NAL, SUL, CT, PB, AMI, FEP, MEM, LEV, DOX) did not have enough samples in one class to allow cross-validation and SMOTE and so were not taken further. Two antibiotics (CAZ and AMC) were further discarded since no predictors passed the feature selection stage (no features were significantly associated with the AMR profiles using a chi-squared test). This resulted in 11 antibiotic models (AZM, CAZ-C, CFX, CHL, CIP, CTX, CTX-C, GEN, KAN, MIN, STR) for the classification, each using between 1 and 23 predictors (ARGs).

To ensure robustness, all antibiotic models were trained and tested against a panel of five classifiers and two meta-methods. The performance was measured in 30 runs of nested cross-validation, with the performance metrics given as the mean of all runs. To verify which classifier performed better out of the seven ML methods, a Friedman F-test with a *post-hoc* Nemenyi test was used (see Supplementary Material and Fig. S8). The logistic regression classifier had the best rank for the AUC metric. Therefore, the logistic regression classifier was selected for the final performance metrics in this study.

Nine (AZM, CFX, CHL, CIP, CTX, KAN, MIN, and STR) of the 11 antibiotic models achieved an AUC score higher than 0.90, with CAZ-C achieving an AUC of 0.87 and only gentamycin achieving an AUC score below 0.80. GEN and CAZ-C had some of the lowest numbers of features with one and four features, respectively. The precision was also high for most antibiotics with values ranging from 0.81–0.96 and five models having a precision over 0.90. The CFX model had the highest AUC score (0.98), it also achieved an accuracy of 0.92, sensitivity of 0.94, specificity of 0.90, and precision of 0.91 (Supplementary Table 9 and Fig. 6B).

To verify if the number of samples for each antibiotic model was large enough for the test set to be representative (i.e. not overtrained), we employed a wrapper backward selection (WBS) to compare training and testing performance [83]. At each run of the WBS, the number of samples is reduced and 5 runs of NCV with logistic regression as the main classifier were used to calculate the average performance in terms of the AUC (see Supplementary Material). We found that despite the small number of samples, the NCV and SMOTE approaches compensated and hence the number of samples was sufficient to train and test the antibiotic models we chose to proceed with (see Supplementary Material and Fig. S9).

An undirected graph (Fig. S10) was created using NetworkX [76] to visualize the ARGs selected by the ML framework (Fig. 6A). These ARGs were the most significantly correlated with the antimicrobial resistance/susceptibility profiles of *E. coli* in broiler chicken faeces. The ARGs belonged to the following antibiotic classes: aminoglycosides (*n* = 13), beta-lactams (*n* = 8), MLSB (*n* = 9), amphenicol (*n* = 5), MDR (*n* = 5), tetracycline (*n* = 4), trimethoprim and sulphonamide (*n* = 4), nucleoside (*n* = 2), peptide (*n* = 1) and glycopeptide (*n* = 1). Five multidrug-resistant genes (ARGs classified by the CARD database as conferring resistance to multiple classes of antibiotics) were selected as relevant features, when generating the predictive models to classify the resistance vs susceptibility profiles of the beta-lactam antibiotics, with two of these genes (*efmA* and *kpnH*) also selected by other antibiotic ML models (ciprofloxacin for *efmA* and streptomycin, kanamycin and chloramphenicol for *kpnH*). MLSB genes were also associated with beta-lactam antibiotics. Of the seven beta-lactam genes selected, five were associated with beta-lactam antibiotics, but two were not, with *CTX-M-3* linked only to ciprofloxacin and *CTX-M-14* linked only to streptomycin. By combining metagenomics data with culture-based methods using ML, we observed that 52 specific ARGs present in the animal gut resistome correlated with the AMR profiles of cultured *E. coli* samples, obtained from the same gut samples. Of these 52 ARGs selected by the ML, only 17 were also found in the WGS of the *E. coli* isolates [24]. Therefore, 35 of these 52 genes (67%) are likely to have originated either from uncultured strains of *E. coli* or from other species.

### ARG diversity of chicken faeces samples changes with local temperature and humidity

To investigate whether the genes associated with AMR resistance/susceptibility profiles on the farm (those selected by the feature selection step in the ML pipeline) were changed or impacted by environmental conditions, we explored the correlation of two environmental factors, temperature and humidity, with broiler chicken gut ARG carriage. We measured the six-hourly temperature and humidity in the barns and averaged them over the seven preceding days before collection for each of the four sample collection dates (T1, T2, L1, and L2) over two production cycles, spring and summer (Fig. S11 and Supplementary Table 10). By performing a linear least-square analysis, using the ARG presence/absence in the 56 broiler chicken faeces samples as independent variables, (Materials and Methods and Fig. 6C), we found ARGs that were significantly correlated (had a slope significantly different from 0 according to the *t*-test) to the dependent variables, temperature and humidity, which were considered separately (Supplementary Table 11 and Fig. S12). We found genes belonging to aminoglycoside, amphenicol, beta-lactam, MLSB, nucleoside, tetracycline, and trimethoprim classes as well as MDR genes, significantly correlated with temperature and humidity (slope significantly different from 0 using a *t*-test, *p* value < 0.05). Of these, five genes were correlated with humidity (slope significantly different from 0 using a *t*-test, *p* values < 0.0001): *CTX-M-65*, *ermA*, *optrA*, *APH (3*′*)-IIa*, and *dfrA1*. Similarly, we found that three genes were correlated with temperature (slope significantly different from 0 using a *t*-test, *p* values < 0.0001): *APH (3*′*)-IIa*, *SAT-1*, and *dfrA1*.

As the associations between ARGs and temperature/humidity could also have been due to antibiotic usage changes between the two production cycles, we tested this possibility. In both production cycles, broiler chickens were dosed with aminoglycosides before the first timepoint, T1 and L1 (Supplementary Table 12). Additionally, spring broiler chickens were dosed with florfenicol, and summer broiler chickens were dosed with amoxicillin. Considering antibiotic classes, we calculated the number of ARGs in each class for each of our 56 broiler chicken samples. We compared whether the number of ARGs in each class varied between the two production cycles, using a Mann-Whitney U test, with Holm correction. The number of MLSB, beta-lactam, and diaminopyrimidine genes significantly differed between the two cycles (Holm correction, *p* value threshold 0.01) with, on average, more ARGs from all three classes present in the summer cycle compared to the spring cycle. Before correcting for multiple comparisons, a further two antibiotic classes were also significant, aminoglycoside and rifamycin. As amoxicillin was given in only the summer production cycle, the usage of this antibiotic may be a confounding factor in the regression analysis of the beta-lactam genes. After correction, the number of aminoglycoside and amphenicol genes present in each sample was not found to significantly differ between the two production cycles, hence we do not observe a statistically significant effect of antibiotic usage of these two antibiotic groups on the number of ARGs found in these two groups. However, although we do not observe a statistically significant effect on the mean number of ARGs, we cannot rule out any effect on either the presence or diversity of ARGs.

## Discussion

In this work, we conducted a longitudinal study on a poultry farm and a connected slaughterhouse in China. We dissected patterns of similarities and differences at a larger scale involving the whole resistome and bacterial taxa distributions between workers and animals as well as, at a finer scale, searching for mobile genetic elements and clinically relevant ARGs. By using an ML-powered approach, we have shown that specific ARGs present in the animal gut resistome correlate with the resistance phenotypes of *E. coli* isolates also present in the animal gut, and the presence of a subset of these ARGs was correlated with environmental temperature and humidity within the animal’s housing.

When considering the microbiota and resistome, separation of bacterial species composition (i.e., type and abundance) and ARG presence-absence profiles was observed across humans, chickens, carcasses, and soil. The separation that we observed agrees with existing literature showing a difference between the human and livestock microbiomes from households living in close contact with backyard livestock [84]. This result is unsurprising since NMDS is a pairwise comparison of the similarity and dissimilarity of the ARGs and species composition in each sample. Differences at this scale of analysis in the chicken and human microbiota and resistome are to be expected as the gut composition is influenced by many host factors, including age, diet, environment, and genetics [85–87], all of which differed between the humans and chickens in our study. However, on an individual basis, many species and ARGs were found in common between samples from different hosts.

When we looked on a finer scale at individual microbes and ARGs, we observed that 14% of species and 76% of ARGs found in humans were also present in chickens. Case-control studies have shown that the gut microbiome of farmworkers and those in close contact with livestock is altered compared to controls without such contact [88, 89]. Our results, without controls, could not assess the origin of these similarities, but instead provided a snapshot of the status of human and chicken microbiomes in this environment. Eleven clinically relevant ARGs, of the 73 previously annotated [44], were found in the same mobile ARG patterns in both hosts. This result was in agreement with a previous study based on pig farms in China, which found that of the 120 medically relevant ARGs they considered, 41% were present in both human and environmental samples (including pig faeces) and that many of those were associated with MGEs [25]. Hu et al. [43] compared the gut metagenome of four chicken samples from suburban farms in Beijing (China) to reference gut microbiome genes from the Chinese Gene Catalog. They found nine of the 73 clinically relevant ARGs we analyzed, present as mobile ARGs in both their chicken and human datasets, which is similar to our result of eleven. Five of these nine ARGs were also found to be present in humans and chickens in our study. Again, without controls, the causes and directionality of these similarities cannot be ruled out, but it is suggestive of an interconnectedness of these environments (humans and chickens), which could, for example, be due to a common source. Some papers have described a high correlation between MGE presence and ARG presence [90, 91], which may suggest, as discussed by others [1], that when investigating AMR, that focussing on individual ARGs and MGEs, may be more relevant than bacterial lineages.

Narrowing down to strain level comparison, we found six chicken MAGs of the 566 constructed that clustered at 99% ANI with human samples. When the 566 MAGs were compared to the public databases, 507 were novel. The amount of similarity we observed, at this high threshold, is comparable to a previous study [58] showing that 9 of 469 chicken caecal MAGs were present in public databases. When focusing only on the indicator species *E. coli* however, phylogenetic analysis of microbial strains suggested that clusters of similar *E. coli* strains (with a normalized phylogenetic distance of no more than 0.1) were present between human and chicken samples, possibly indicating either contamination between hosts or a common source of bacterial contamination, though we do not, in this paper, address the cause of these clusters. In a WGS analysis of *E. coli* strains isolated from chicken faeces, carcasses, caecal droppings and anal swabs, and human faeces, hands, nasal swabs as well as water, soil and feed, we also saw clustering of human and chicken samples [24]. A subset of these samples, 46 chicken faeces samples and 30 human faeces samples, were in common between the present study and Peng et al. [24]. Compared with the metagenome data we study here, the whole genome study [24] found stronger evidence of clustering between isolates from different hosts, however, hotspots of similarities were found between samples from livestock and the nose and hands of humans. However, nose and hands were excluded from this study as there were no corresponding metagenome samples. On the other hand, the whole genome analysis [23] could not consider the resistome from which the *E. coli* was taken and hence may have missed important ARGs [38].

Then, by combining metagenomics data with culture-based methods using ML, we observed that 52 specific ARGs present in the animal gut resistome correlated with the AMR profiles of cultured *E. coli* isolates, obtained from the same gut samples. Comparing these ARGs to the ones found in the *E. coli* isolates [24] only 17 were found in both studies. Therefore, 35 of these 52 genes (67%) are likely to have originated either from uncultured strains of *E. coli* or from other species in the microbiome and would have been missed with conventional whole genome approaches alone. This suggests the importance of the microbial reservoir to resistance phenotypes and the relevance of monitoring them in parallel, as well as the development of methods able to detect these associations with a certain level of accuracy. In the literature, there are several examples of ML methods applied to metagenomic analysis: to draw microbiome comparisons between different sample types (e.g., healthy vs diseased individuals, different environments, etc.) using statistical methods such as principal component analysis [92–94]; to classify healthy and diseased individuals for a range of conditions including liver cirrhosis, colorectal cancer and irritable bowel disease [95]; to correlate individual bacteria and the difference in abundances of various genetic markers which are commonly analyzed within clinical applications (e.g. the change in the microbiome correlated to recurrent infections) [96–98]; and to search for associations of gut microbiome composition with the presence and potential colonization of resistant bacteria [99, 100]. However, to our knowledge, this is the first time that phenotypic resistance of indicator species has been correlated with the resistome using the supervised ML methods considered in this work.

Finally, in this work, regression analysis indicated correlations between the ARG presence-absence from the chicken samples and the temperature and humidity data inside the barn, for a subset of the 52 ARGs that were significantly correlated with AMR phenotypes. Although these differences may be the result of batch variations [101] or antibiotic usage, our results suggest they could also be directly due to seasonal temperature and humidity changes, for which significant evidence has been seen in other studies [102–104]. Two of the five genes that positively correlated with humidity were MLSB genes. Similar results were shown in a recent study where MLSB gene presence was positively correlated with soil temperature and moisture in different wetlands in China [105]. MacFadden et al. [106] also recently showed that climate may contribute to population-level increases in antibiotic resistance. It is possible that higher temperatures may facilitate horizontal gene transfer [107], though these results could also be the consequence of increased microbial abundance. Seasonal oscillations of antibiotic resistance genes, especially in natural environments, have been previously reported [108]. To what extent the broiler chicken gut ARGs were affected by these two co-dependent environmental factors, temperature, and humidity, has not been fully addressed here and would need to be explored with a larger sample size accounting for confounding factors.

We acknowledge that this study had several limitations. Firstly, the sampling of abattoir workers and their households on the day of slaughter was not ideal as any potential changes in the gut microbiome as a result of contact with the chickens and/or carcasses would not yet be reflected in the gut. Whilst hand samples were originally included in the study design, these did not obtain enough DNA for sequencing. Ideally, multiple samples taken both before and after slaughter would be better for observing changes, however, given the nature of poultry meat processing in the slaughterhouse, this may be infeasible as poultry is being continuously processed from multiple farms. Secondly, the results shown here may be limited by the mapping of the metagenome samples to only one cultured *E. coli* strain per sample. Whilst many AMR surveillance studies of commensal gut bacteria also use the approach of taking only one isolate per sample [109–112], a mixed *E. coli* population in faeces is highly likely [113], and isolating multiple strains per sample could give a more reproducible result and could be incorporated in a larger study. Thirdly, in this study, soil and carcass, samples had a low sequencing depth and consequent poor assembly quality, which limited any conclusions of these samples compared to other sample types; however, the depth and quality of chicken and human faeces samples were robust.

More generally, whilst metagenomic techniques may have some advantage over conventional approaches due to their capacity to detect and quantify thousands of resistance genes present in the bacterial microbiome of each sample, some limitations need to be appreciated when applying these methods [114]. Metagenomic techniques typically have lower granularity to appreciate changes within specific genes or strains, compared to whole genome approaches which may bias against the detection of rare ARGs and ARBs [32, 93, 114, 115]; hence caution is needed. Also, metagenomics results are sensitive to the sampling matrix, DNA extraction procedures, and library preparation [116], hence results can be less comparable across different sample types and studies, than culture-based approaches, and require normalization. Finally, and perhaps most challenging, it can be difficult to link ARGs in the resistome back so the microbial species they originated from [117]. Therefore, the use of metagenomics still requires novel tools and information to accurately predict AMR and identify the organisms carrying the ARGs [118].

Harmonized ML protocols and bioinformatic tools for the association of the resistome and observable resistance phenotypes are not readily available as the performance indicators such as sensitivity, specificity, and reproducibility of the methods need yet to be improved [32]. Also, it is currently not feasible to implement these methods globally, especially in LMICs. However, this study should be considered a proof-of-principle to be further investigated and validated with larger samples and different geographical areas. After validation, by applying our method at larger spatial, geographical, and temporal scales, this protocol might offer the possibility to inform novel solutions for epidemiological monitoring suitable for deployment in LMICs. Especially in consideration of how this knowledge and methods could be used to mitigate AMR within the landscape of increasing adoption of sensing/monitoring technologies, digitalization in livestock farming [119–121], and the adoption of the latest technologies in ML and big data mining to implement precision poultry farming [122, 123].

## Supplementary information

Supplementary Material

Supplementary Table 1

Supplementary Table 2

Supplementary Table 3

Supplementary Table 4

Supplementary Table 5

Supplementary Table 6

Supplementary Table 7

Supplementary Table 8

Supplementary Table 9

Supplementary Table 10

Supplementary Table 11

Supplementary Table 12

Supplementary Table 13

## Data Availability

The metagenomic sequencing data supporting the conclusions of this article are available in the NCBI under Bioproject accession number PRJNA678871. The code used in this study is available in the following GitHub repository: https://github.com/tan0101/MGS_ISME2022.
