# Supplementary material for: Dissecting microbial communities and resistomes for interconnected humans, soil, and livestock
Source: ISME J. 2022 Sep 23;17(1):21–35. doi: 10.1038/s41396-022-01315-7 (PMC9751072; doi:10.1038/s41396-022-01315-7)
Supplement: Supplementary file 1 — Supplementary Material [file 41396_2022_1315_MOESM1_ESM.docx]

Supplementary Material for

**Dissecting microbial communities and resistomes for interconnected humans, soil, and livestock.**

Alexandre Maciel-Guerra^1‡^, Michelle Baker^1‡^, Yue Hu^1‡^, Wei Wang^2^, Xibin Zhang^3^, Jia Rong^3^, Yimin Zhang^4^, Jing Zhang^2^, Jasmeet Kaler^1^, David Renney^5^, Matthew Loose^6^, Richard D Emes^1^, Longhai Liu^3^, Junshi Chen^2^, Zixin Peng^2*^, Fengqin Li^2*^, and Tania Dottorini^1*^

^‡^Co-first authors

*corresponding authors

**Contents:**

**Supplementary Methods**

**Supplementary note on MAGs novelty**

**Supplementary note on shared MAGs**

**Supplementary note on machine learning**

**Supplementary Figures 1-12**

**List of Supplementary Tables**

**Supplementary Methods**

*Antibiotic susceptibility testing of E. coli isolates*

For each sample, where possible, *E. coli* strains were cultured as indicator organisms. 1g sample of faeces and soil was vortexed with 9 mL of sterile buffered peptone water tube (BPW; Luqiao Inc., Beijing, China) for 1 min. Broiler carcass sponge samples were homogenised with 10 mL BPW for 1 min in a stomacher bag. Approximately 1 mL was added to 9 mL *E. coli* (EC) broth (Luqiao Inc.) and incubated at 37℃ for 16-20 h. A loopful of these solutions was then streaked onto an eosin-methylene blue (EMB) agar and MacConkey (MAC) Agar (Luqiao Inc.) and incubated at 37°C for 18-24 h. Typical *E. coli* colonies were counted and subsequently characterized by Bruker MALDI Biotyper (Germany).

The antimicrobial susceptibility testing was carried out on the cultured *E. coli* isolates. Antimicrobial susceptibility to a panel of agents was determined by broth microdilution and interpreted according to the criteria based on the Clinical & Laboratory Standards Institute (CLSI) interpretive criteria (CLSI 2009). The minimum inhibitory concentrations (MIC) of 28 antimicrobial compounds were measured for the *E. coli* isolates: ampicillin (AMP), ampicillin/sulbactam (AMS), tetracycline (TET), chloramphenicol (CHL), trimethoprim/sulfamethoxazole (SXT), cephazolin (CFZ), cefotaxime (CTX), ceftazidime (CAZ), cefoxitin (CFX), gentamicin (GEN), imipenem (IMI), nalidixic acid (NAL), sulfisoxazole (SUL), ciprofloxacin (CIP), amoxicillin/clavulanic acid (AMC), cefotaxime/clavulanic acid (CTX-C), ceftazidime/clavulanic acid (CAZ-C), polymyxin E (CT), polymyxin B (PB), minocycline (MIN), amikacin (AMI), aztreonam (AZM), cefepime (FEP), meropenem (MEM), levofloxacin (LEV), doxycycline (DOX), kanamycin (KAN), streptomycin (STR). The resistance/susceptibility profiles for each isolate were calculated (summarised in Supplementary Table 1). *E. coli* ATCC™25922 was used as a control bacterium for these experiments.

*Bioinformatics Analysis*

To construct the microbiome of the samples, assembly of metagenome sequencing data was performed separately for the different sample sources (human, chicken, carcasses and environment) using binning and dereplication pipelines as previously described(1, 2). MEGAHIT (3) software v1.1.2 was used to assemble the sequences. Single sample assemblies for all 137 samples (broiler, human and environment) were generated with MEGAHIT default parameters. Co-assemblies were generated for both the broiler carcass and broiler faeces sample groups, each with MEGAHIT setting parameters of “--continue --kmin-1pass --min-contig-len 1000” as previously used on co-assemblies (1). Filtered contigs (>2000 bp) were mapped to single assemblies and co-assemblies using BWA MEM (4) and SAMtools (4) to produce the BAM files. METABAT2(4) was used to obtain the depth of coverage and to reconstruct genome bins on both single-sample assemblies and co-assemblies, considering coverage values and with the parameters “--minContigLength 1500”. Bins from single and co-assemblies were combined to dereplicate at 99% average nucleotide identity (ANI) using dRep (4) (with the parameters dereplicate_wf -comp 80 -con 10), with each Metagenome-Assembled Genome (MAG) being taxonomically equivalent to a microbial strain. Bins were also dereplicated at 95% ANI with the parameters “dereplicate_wf -comp 80 -con 10 -sa 0.95” to calculate the number of species represented within identified MAGs. The completeness and contamination of all bins were assessed using CheckM (5) with the options “lineage_wf, -t 16, -x fa” and filtering for completeness ≥ 80% and contamination ≤ 10%. The taxonomic trees were visualised using Graphlan(6). Genomes were assigned taxonomy through GTDB-Tk v1.0.2 (7) as novel strains if the ANI output from GTDB-Tk was < 99%. Genomes were determined as novel species if the ANI output from GTDB-Tk was < 95%, following the pipeline of Glendinning et al.(1) . Where MAGs were not assigned a genus by GTDB-Tk, they were checked at 60% AAI using CompareM (<https://github.com/dparks1134/CompareM>). If they were still unable to cluster with known MAGs, they were determined to be novel genera.

*Machine Learning hyper-parameter choice*

For the classifiers, the following set of values are employed for the hyper-parameters:

• Logistic Regression: inverse of regularization strength C = [0.001, 0.01, 0.1, 1, 10, 100, 1000].

• Linear SVM: penalty parameter of the hinge loss error C = [0.001, 0.01, 0.1, 1, 10, 100, 1000].

• Random Forest and Adaboost: Number of estimators = [2, 4, 8, 16, 32, 64].

• Non-linear SVM with RBF kernel: γ(RBF kernel coefficient) = [0.0001, 0.001, 0.01, 0.1] and C (L2 penalty parameter) = [0.001, 0.01, 0.1, 1, 10, 100, 1000]

• XGBoost: Number of estimators = [2, 4, 8, 16, 32, 64] and learning rate = [0.001, 0.01, 0.1, 1]”

**Supplementary note on MAGs novelty**

From the taxonomic identification, all 566 broiler chicken faeces MAGs were identified as bacteria. The most dominant phylum was *Firmicutes* containing *Firmicutes_A* (n=455), *Firmicutes* (n = 79) and *Firmicutes_B* (n =1: family UBA7702). This is followed by *Actinobacteriota* (n = 9), *Bacteroidota* (n = 7), *Proteobacteria* (n = 7), *Cyanobacteria* (n = 6: order *Gastranaerophilales*), *Campylobacterota* (n = 1: *Helicobacter_D pullorum*), *Verrucomicrobiota* (n =1: *Akkermansia muciniphila*). All members of *Firmincutes_A* belonged to the class *Clostridia*, which includes the orders *Oscillospirales* (n = 175), *Lachnospirales* (n = 140), *4C28d-15* (n = 113), *Christensenellales* (n = 10), *Peptostreptococcales* (n =6), *TANB77* (n = 3), *Clostridiales* (n = 2), *Monoglobales* (n = 2), *UBA1212* (n = 2), *Eubacteriales* (n = 1) and one undefined MAG. All members of *Firmicutes* belonged to the class *Bacilli*, which include the orders RF39 (n = 40), *Lactobacillales* (n = 22), *Erysipelotrichales* (n = 13), RFN20 (n = 2), *Exiguobacterales* (n = 1), and *Acholeplasmatales* (n = 1). The *Actinobacteriota* were divided into two classes, *Actinobacteria* (n = 1: genus *Rothia*) and *Coriobacteria* (n = 8: all from family *Eggerthellaceae*). The family *Eggerthellaceae* contains 7 strains of *Rubneribacter badeniensis* and 1 strain of *Gordonibacter pamelaeae*. All members of *Bacteroidota* belonged to the order *Bacteroidales*, which included two families *Bacteroidaceae* (n =3: genus *Bacteroides*) and *Rikenellaceae* (n =4). The family *Rikenellaceae* contains three genera including *Alistipes* (n = 2), *Tidjanibacter* (n =1: *Tidjanibacter inops*) and *Rikenella* (n =1: *Rikenella microfusus*). The *Proteobacteria* were divided into two classes, *Alphaproteobacteria* (n = 1: species *Azospirillum* sp. 47_25 (CAG-495)) and *Gammaproteobacteria* (n = 6). The *Gammaproteobacteria* contain two genus *Enterobacter* (n = 1) and *Escherichia* (n = 5).

We compiled a dataset comprising our MAGs and the sequenced genomes previously identified in chickens from Medvecky *et al.* (8) and Carrasco *et al.* (9). Of the MAGs that showed greater than 95% ANI with the existing sequenced genomes, our MAGs included 1 novel strain of *Akkermansia muciniphila*, 5 novel strains of *Anaeromassilibacillus senegalensis* An250, 1 novel strain of *Anaerofilum* sp. An201, 7 novel strains of *Blautia sp*. (6 of *Blautia producta* An81 and 1 of *Blautia schinkii* An249), 1 novel strain of *Clostridiales* bacterium CHKCI006, 4 novel strains of *Drancourtella sp.* (3 An57 and 1 An210), 1 novel strain of *Enterococcus cecorum*, 2 novel strains of *Erysipelatoclostridum sp.* (1 *Erysipelatoclostridum spiroforme* and 1 An173), 3 novel strains of *E.coli*, 5 novel strains of *Eubacteriaceae* bacterium CHKCI004, a novel strain of *Eubacterium* sp. An11, 3 novel strains of *Flavonifactor* spp. (2 An112, 1 An10), 2 novel strains of *Gemmiger* sp. (1 An120 and 1 An50), 7 novel strains of *Lachnoclostridium* sp. (1 An118, 1 An138, 1 An14, 2 An196, 1 An298, 1An76), 5 novel strains of *Massiliomicrobiota* sp. ( 4 An134 and 1 An13), 4 novel strains of *Pseudoflavonifractor* spp. (1 An184 and 2 An44).

By combining these 566 MAGs with the 469 caecal MAGs characterised by Glendinning *et al.*(1) with dereplication at 99% ANI, a total of 952 MAGs remained including 445 MAGs from ceca MAGs and 507 MAGs from this study with novel putative strains. Specifically, 2 novel strains of *Aerococcus urinaeequi*, 1 novel strain of *Alistipes finegoldii*, 1 novel strain of *Bacteroides clarus*, 1 novel strain of *Bacteroides fragilis*, 1 novel strain of *Bacteroides xylanisolvens*, 1 novel strain of *Enterococcus faecalis*, 1 novel strain of *Enterococcus hirae*, 1 novel strain of *Enterococcus gallinarum*, 2 novel strains of *Firmicutes* bacterium spp. (1 CAG:631, 1 CAG:988, 3 novel strains of *Merdimonas faecis*, 1 novel strain of *Faecalitalea sp. Marseille-P3755*, 1 novel strain of *Rikenella microfusus*, 7 novel strains of *Rubneribacter badeniensis*, 3 novel strains of *Ruminococcaceae* bacterium spp. (2 UBA1816 and 1 UBA1375), 1 novel strain of *Subdoligranulum sp.* CAG:314 and 1 novel strain of *Weissella paramesenteroides* were further characterised only from this study. In addition, 10 novel strains of *Lactobacillus spp.* which have been suggested as potential probiotics in chickens were also characterised. This includes 3 novel strains of *Lactobacillus aviaries*, 1 novel strain of *Lactobacillus crispatus*, 1 novel strain of *Lactobacillus johnsonii*, 1 novel strain of *Lactobacillus oris*, 1 novel strain of *Lactobacillus pontis*, 2 novel strains of *Lactobacillus reuteri*, 1 novel strain of *Lactobacillus salivarius*, 1 novel strain of *Lactobacillus timonensis* and 1 novel strain of *Lactobacillus vaginalis*.

The identified 323 novel species across different taxonomies: included 270 species of class *Clostridia*, 47 species of class *Bacilli*, 1 novel species identified to family UBA7702 belonging to class *Dehalobacteriia*, 4 novel species identified to order *Gastranaerophilales* belonging to class *Vampirovibrionia*, and 1 novel species identified to genus *Rothia* belongs to class *Actinobacteria*. For the novel species identified in class *Bacilli*, 38 MAGs belong to order RF39, 1 novel species identified to order *Exiguobacterales*, 1 novel species identified to family CAG-631, 1 novel species identified to family CAG-307, 2 novel species identified to genus *Lactobacillus*, 2 novel species identified to genus *Merdibacter*, 1 novel species identified to genus *Holdemania* and 1 novel species identified to genus *Erysipelatoclostridium*. For the novel species identified in class *Clostridia*, 100 MAGs belong to order *Oscillospirales*, 87 MAGs belong to order *Lachnospirales*, 57 MAGs belong to order 4C28d-15, 10 MAGs belong to order *Christensenellales*, 6 novel species belong to order *Peptostreptococcales*, 3 novel species identified to genus TANB77 (1 of UBA7001, 1 of CAG-273 and 1 of CAG-354), 2 novel species belong to order UBA1212, 2 novel species belong to order *Monoglobales*, 1 novel species identified to family *Anaerofustaceae* and 1 novel species identified to genus *Clostridium*.

For the human faeces MAGs, 573 originated from bacteria and 1 MAG originated from archaea, belonging to the species *Methanobrevibacter A smithii*. The majority of the bacterial MAGs were able to be identified to species (474 out of 573), 79 identified to genus, 18 identified to family, and 3 identified to order. The most dominant phylum was *Firmicutes* containing *Firmicutes_A* (n=382), *Firmicutes* (n = 33), *Firmicutes_C* (n =8) and *Firmicutes_B* (n=1: family *Peptococcaceae*). This is followed by *Bacteroidota* (n =76), *Actinobacteriota* (n = 34), *Proteobacteria* (n =21), *Verrucomicrobiota* (n =6), *Cyanobacteria* (n =4: order *Gastranaerophilaceae*), and *Desulfobacterota_A* (n =2: family *Desulfovibrionaceae*). All members of *Firmincutes_A* belonged to the class *Clostridia*, which includes the orders *Lachnospirales* (n = 186), *Oscillospirales* (n = 164), *Christensenellales* (n = 13), Monoglobales (n = 10), *Peptostreptococcales* (n =4), *Clostridiales* (n = 3), *TANB77* (n = 1), and *4C28d-15* (n = 1). All members of *Firmicutes* belonged to the class *Bacilli*, which include the orders *Erysipelotrichales* (n = 14), *RF39* (n = 10), *Lactobacillales* (n = 4), *RFN20* (n = 2), *ML615J-28* (n =2), *Haloplasmatales* (n =1). All members of *Bacteroidota* belonged to the class *Bacteroidia*, which includes the orders Bacteroidales (n = 73) and *Flavobacteriales* (n =3). The *Actinobacteriota* were divided into two classes, *Actinobacteria* (n = 10: order *Actinomycetales*) and *Coriobacteria* (n = 24: *Coriobacteriales*). The *Proteobacteria* were divided into two classes, *Alphaproteobacteria* (n =5: family *CAG-239*) and *Gammaproteobacteria* (n = 16). The *Gammaproteobacteria* contain two orders *Enterobacterales* (n = 6) and *Burkholderiales* (n = 10).

Of the MAGs that showed greater than 95% ANI with the existing sequenced genomes, our MAGs included, in phylum Actinobacteriota, 6 novel strains of Adlercreutzia equolifaciens, 2 novel strains of *Bifidobacterium adolescentis*, 1 novel strain of *Bifidobacterium dentium*, 1 novel strain of *Bifidobacterium longum*, 1 novel strain of *CAG-1427 sp.*, 3 novel strain of *Collinsella sp.*, 1 novel strain of *Eggerthella lenta*, 1 novel strain of *Rothia mucilaginosa* and 5 novel strains of *Senegalimassilia anaerobia*. In phylum *Bacteroidota*, our MAGs include 1 novel strain of *Alistipes obesi*, 5 novel strains of *Alistipes onderdonkii*, 2 novel strains of *Alistipes putredinis*, 1 novel strain of *Alistipes senegalensis*, 1 novel strain of *Alistipes ihumii*, 1 novel strain of *Bacteroides intestinalis*, 1 novel strain of *Bacteroides ovatus*, 3 novel strains of *Bacteroides stercoris*, 6 novel strains of *Bacteroides uniformis*, 2 novel strains of *Bacteroides xylanisolvens*, 1 novel strain of *Bacteroides coprocola*, 7 novel strains of *Bacteroides plebeius*, 1 novel strain of *Bacteroides massiliensis*, 1 novel strain of *Bacteroides vulgatus*, 5 novel strains of *Barnesiella intestinihominis*, 1 novel strain of *CAG-1031 sp.*, 3 novel strains of *CAG-279 sp.*, 2 novel strains of *Coprobacter fastidiosus*, 3 novel strains of *Parabacteroides distasonis*, 3 novel strains of *Parabacteroides merdae*, 4 novel strains of *Paraprevotella clara*, 1 novel strain of *Paraprevotella xylaniphila*, 1 novel strain of *Prevotella copri*, 1 novel strain of *Prevotella sp.* and 1 novel strain of *RC9 sp.*. In phylum *Cyanobacteri*, our MAGs include 1 novel strain of *CAG-306 sp.* and 2 novel strains of *Zag111 sp.*. In phylum *Desulfobacterota*, 1 novel strain of *Bilophila wadsworthia* and 1 novel strain of *Desulfovibrio piger* were characterised from MAGs. In phylum *Firmicutes*, our MAGs include 8 novel strains of *Acetatifactor sp.*, 8 novel strains of *Agathobacter faecis*, 7 novel strains of *Agathobacter rectale*, 8 novel strains of *Anaerostipes hadrus*, 14 novel strains of *Blautia sp.*, 10 novel strains of *CAG-417 sp.*, 10 novel strains of *CAG-41 sp.*, 8 novel strains of *Clostridium_Q sp.*, 9 novel strains of *ER4 sp.*, 6 novel strains of *Erysipelatoclostridium*, 17 novel strains of *Faecalibacterium prausnitzii*, 1 novel strain of *Lactobacillus crispatus*, 1 novel strain of *Lactobacillus salivarius* and 9 novel strains of *TF01-11 sp.*. In phylum *Proteobacteria*, our MAGs include 1 novel strain of *Duodenibacillus massiliensis*, 1 novel strain of *Enterobacter cloacae*, 2 novel strains of *Escherichia coli*, 2 novel strains of *Parasutterella sp.*, 1 novel strain of *RF32 sp. 51-20*, 1 novel strain of *RF32 sp. CAG-495*, 1 novel strain of *Sutterella sp. CAG-521*, 3 novel strains of *Sutterella wadsworthensis*. In phylum *Verrucomicrobiota*, our MAGs include 3 novel strains of *Akkermansia muciniphila* and 1 novel strain of *Victivallales bacterium* *species*.

**MAGs similar between sources and ARGs present**

74 MAGs were similar between human and broiler chicken samples carrying an average of 5 ARGs (Supplementary Table 13). Within this group *Escherichia* spp., *Klebsiella* spp. and *Enterobacter* spp. carried considerably more ARGs with on average more than 20 per MAG, whilst other genus carried 5 or less on average. Twelve *E. coli* MAGs were identified amongst the metagenomic samples, four from broiler chicken faeces and eight from human faeces (Supplementary Table 13). No *E. coli* MAGs could be assembled from broiler chicken carcass or soil samples. *E. coli* MAGs carried on average 37 ARGs (range 33-44) with no difference between the number of ARGs in each source (p-value = 0.346, Kruskal-Wallis test).

**Supplementary note on machine learning**

*Comparison of ML classifiers*

To compare the results obtained by the 5 different classifiers and 2 meta-methods used, a Friedman Statistical F-test was employed. The Friedman test FF with Iman-Davenport correction (10) is employed for statistical comparison of multiple classifiers as suggested by Demsar *et al*. (11). First, we rank the algorithms for each dataset separately, i.e., the best algorithm gets ranking 1, the second-best ranking 2, and so on. In the case of ties, average ranks are assigned. Next, we apply the FF and verify if the null hypothesis is rejected. After, the post-hoc Nemenyi test (11) was used to find if there is a single classifier or a group of classifiers that differ in terms of their average rank after the FF test has rejected the null hypothesis that the performance of the comparisons on the groups of data is similar.

For the broiler chicken resistome, with 7 classifiers and 11 antibiotics, the Friedman test is distributed according to the F distribution with 11−1 = 10 and (7−1)×(11−1) = 60 degrees of freedom. The critical value of F(10,60) for α= 0.0001 is 4.4815. For the broiler chicken microbiome, with 7 classifiers and 7 antibiotics, the Friedman test is distributed according to the F distribution with 7−1 = 6 and (7−1)×(7−1) = 36 degrees of freedom. The critical value of F(6,36) for α= 0.0001 is 6.5267. The null hypothesis states that there are no differences between the average ranks of each classifier over the studied antibiotics. For the human resistome, with 7 classifiers and 9 antibiotics, the Friedman test is distributed according to the F distribution with 7−1 = 6 and (7−1)×(9−1) = 48 degrees of freedom. The critical value of F(6,48) for α= 0.0001 is 5.9838. The null hypothesis states that there are no differences between the average ranks of each classifier over the studied antibiotics. The null hypothesis was rejected with a confidence level of 99.99% for the metric AUC when analysing the broiler chicken resistome data, for all the other metrics in the broiler chicken resistome and all the metrics in the broiler chicken microbiome and the human resistome the null hypothesis was not rejected. After, the Nemenyi post-hoc test was performed, and the critical difference diagram was set at 3.179 with a confidence level of 99%. The logistic regression classifier had the best rank for the metrics AUC for the resistome (Fig. S8). Therefore, the logistic regression classifier was selected for this study.

*Sample size validation*

To analyse if the antibiotics studied had enough samples to make the test representative, a wrapper backward selection (WBS) approach in terms of samples was used. i.e. if initially there are *n* samples the model is tested using all *n*-1 possible sample combinations (leaving one out), the worst model performance is identified and the sample that was left out on this performance is removed for the next iteration. This is done until the minimum number of samples (12) is reached for one of the classes. This minimum number of samples is a requirement for the SMOTE approach used in the classification framework.

We first performed a SMOTE approach as a pre-processing step to oversample the minority class and then applied the WBS method to the oversampled data and analysed using 5 iterations of a nested cross-validation with logistic regression as the classifier (Fig. S9). For the classification of *E. coli* AMR profiles using broiler chicken ARG presence-absence data, 4 antibiotics (GEN, CTX-C, CAZ-C and KAN) had a constant drop in both the training and the testing performance (Fig. S9). However, the other 7 out of 11 antibiotics show training and test datasets are representative for the initial number of samples in each antibiotic (i.e. the test and training performance is close together). With a reduced number of samples, the majority of the antibiotics have a decrease in the test fold performance, indicating that a reduced number of samples, in this case, can cause overfitting of the classifier, since the test fold is not representative anymore. Also, it is interesting to notice that for the antibiotics CTX, CFX, CIP, MIN and AZM the performance of the testing set is kept above 0.9 even after the number of samples is reduced below the original number (blue vertical line). The curve of the test performance for all models is reaching an asymptote indicating that increasing sample sizes further would be unlikely to significantly improve model performance. Taken together these results indicate that whilst the number of samples is low the test data is representative of the full data set, hence the performance metrics are robust and reliable.

**Supplementary Figures**

**
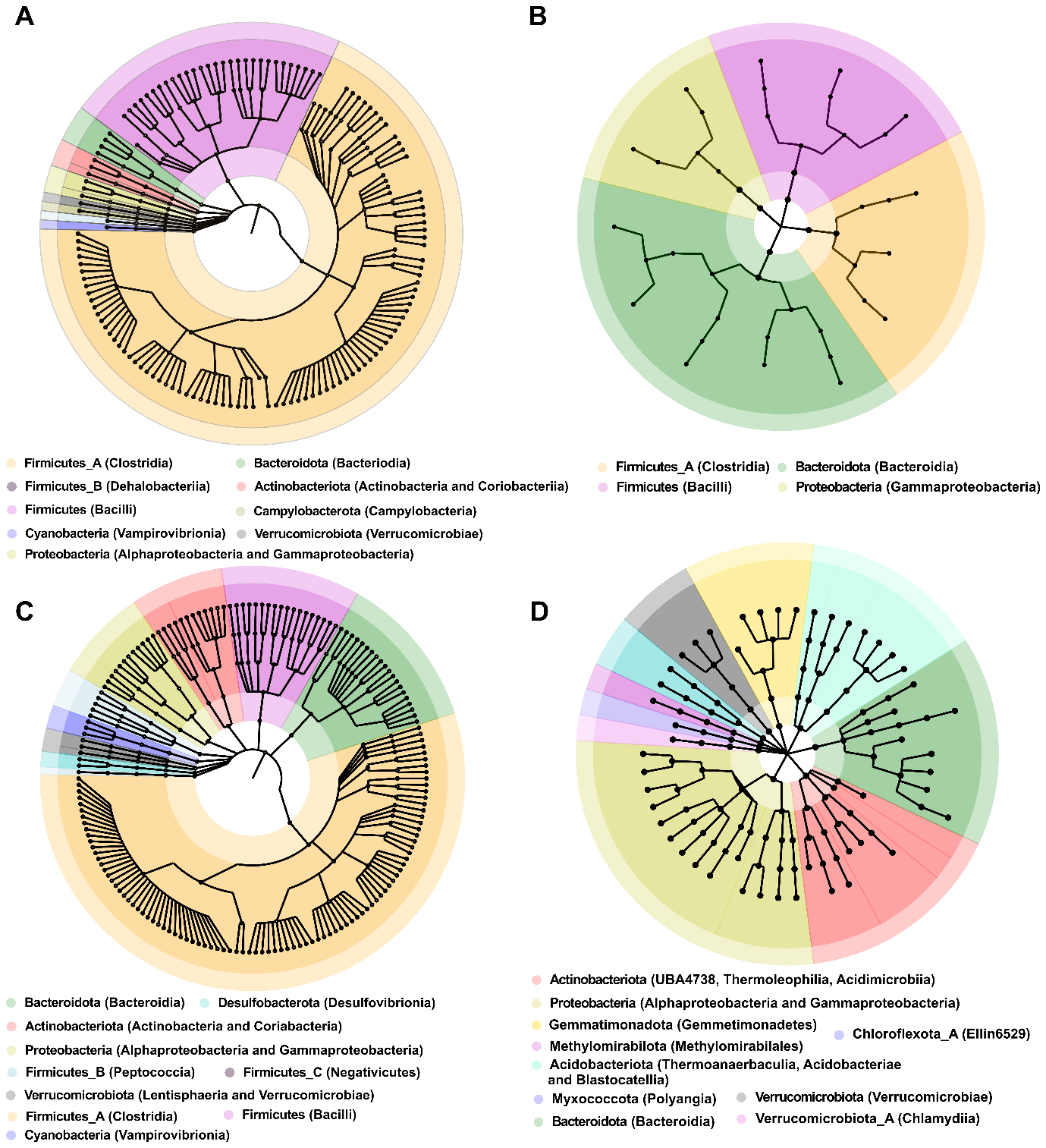
Figure S1. Phylogenetic trees showing the abundance and diversity of the draft microbial genomes from chickens and humans.** Each tree is labelled by taxonomic phylum (with classes given in brackets in a clockwise direction), as defined by GTDB-Tk and NCBI taxonomy. **(A)** Phylogenetic trees representing the draft microbial genomes from broiler chicken faeces samples. The most dominant phylum was *Firmicutes* (n = 535). This is followed by *Actinobacteriota* (n = 9), *Bacteroidota* (n = 7), *Proteobacteria* (n = 7), *Cyanobacteria* (n = 6: order *Gastranaerophilales*), *Campylobacterota* (n = 1: *Helicobacter pullorum*), *Verrucomicrobiota* (n = 1*: Akkermansia muciniphila*). **(B)** Phylogenetic trees representing the draft microbial genomes from broiler chicken carcass samples. The most dominant phylum was *Bacteroidota* (n = 5). **(C)** Phylogenetic trees representing the draft microbial genomes from human faeces samples. The most dominant phylum was *Firmicutes* (n = 424). This is followed by *Bacteroidota* (n = 76), *Actinobacteriota* (n = 34), *Proteobacteria* (n = 21), *Verrucomicrobiota* (n = 6), *Cyanobacteria* (n = 4: order *Gastranaerophilaceae*) and *Desulfobacterota_A* (n = 2: family *Desulfovibrionaceae*). **(D)** Phylogenetic trees representing the draft microbial genomes from soil samples. The most dominant phylum was *Proteobacteria* (n = 14).

**
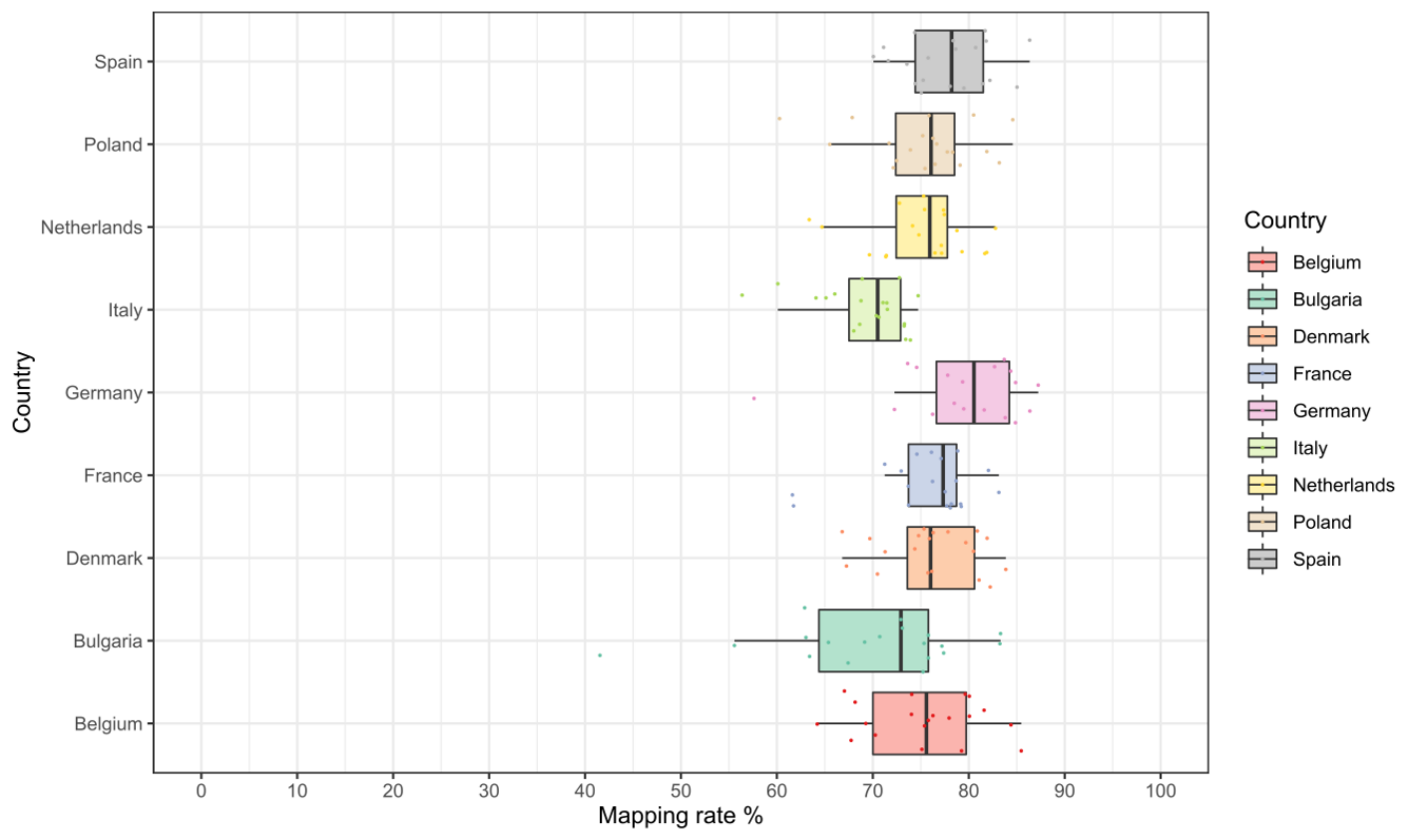
**

**Figure S2. Boxplot of read mapping rates for the broiler chicken samples from a pan-EU study using constructed 566 MAGs in this study.** Adapted from Glendinning et al.^12^

**
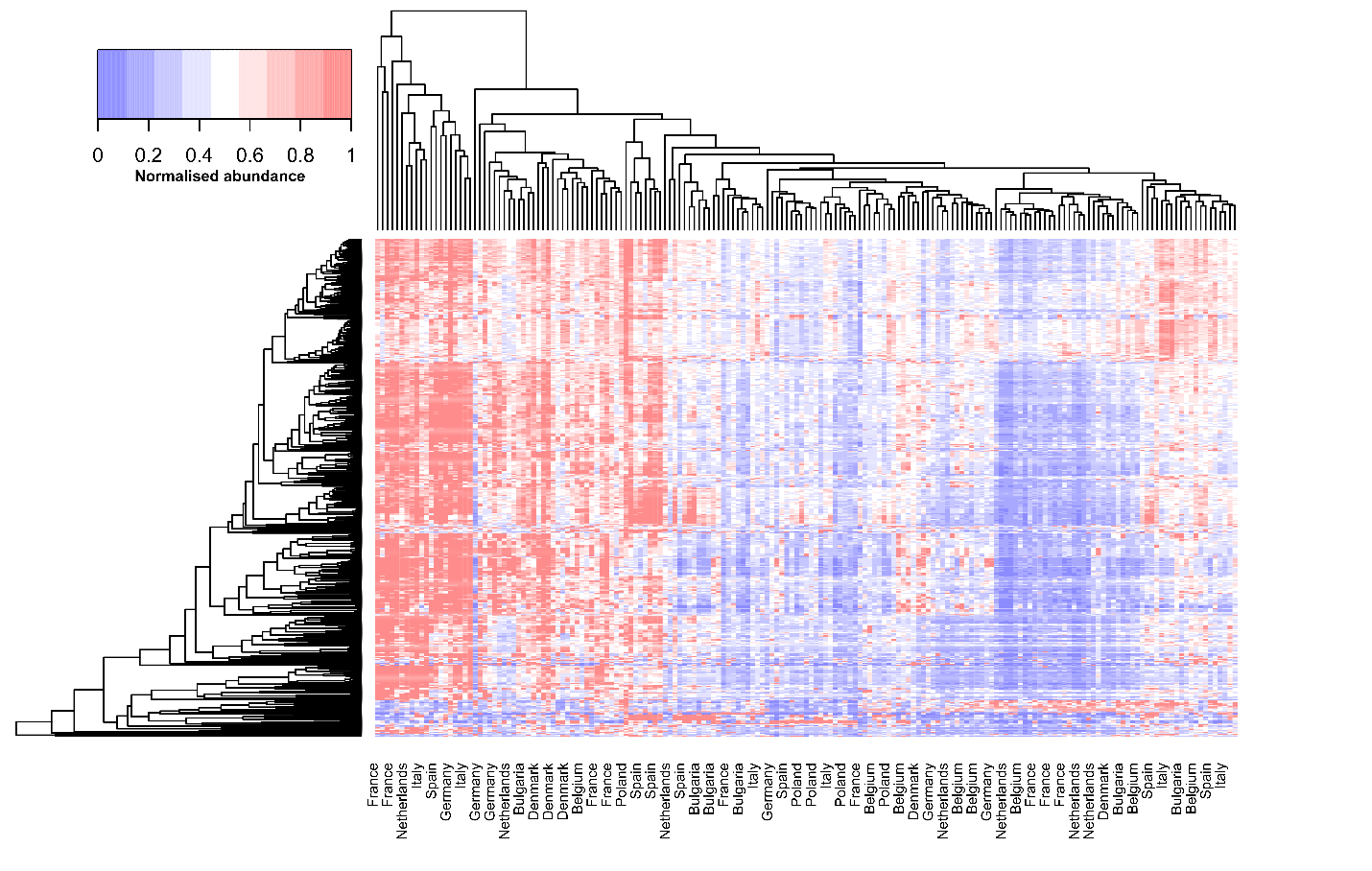
**

**Figure S3 Heatmap of the abundance for the reads mapping of the EU samples to 566 broiler chicken faecal MAGs.** Rows represent each of the 556 broiler chicken faecal MAGS from this study whilst columns represent data from pan-EU study^15^**.** Data are scaled by row. Adapted from Glendinning et al.^12^

**
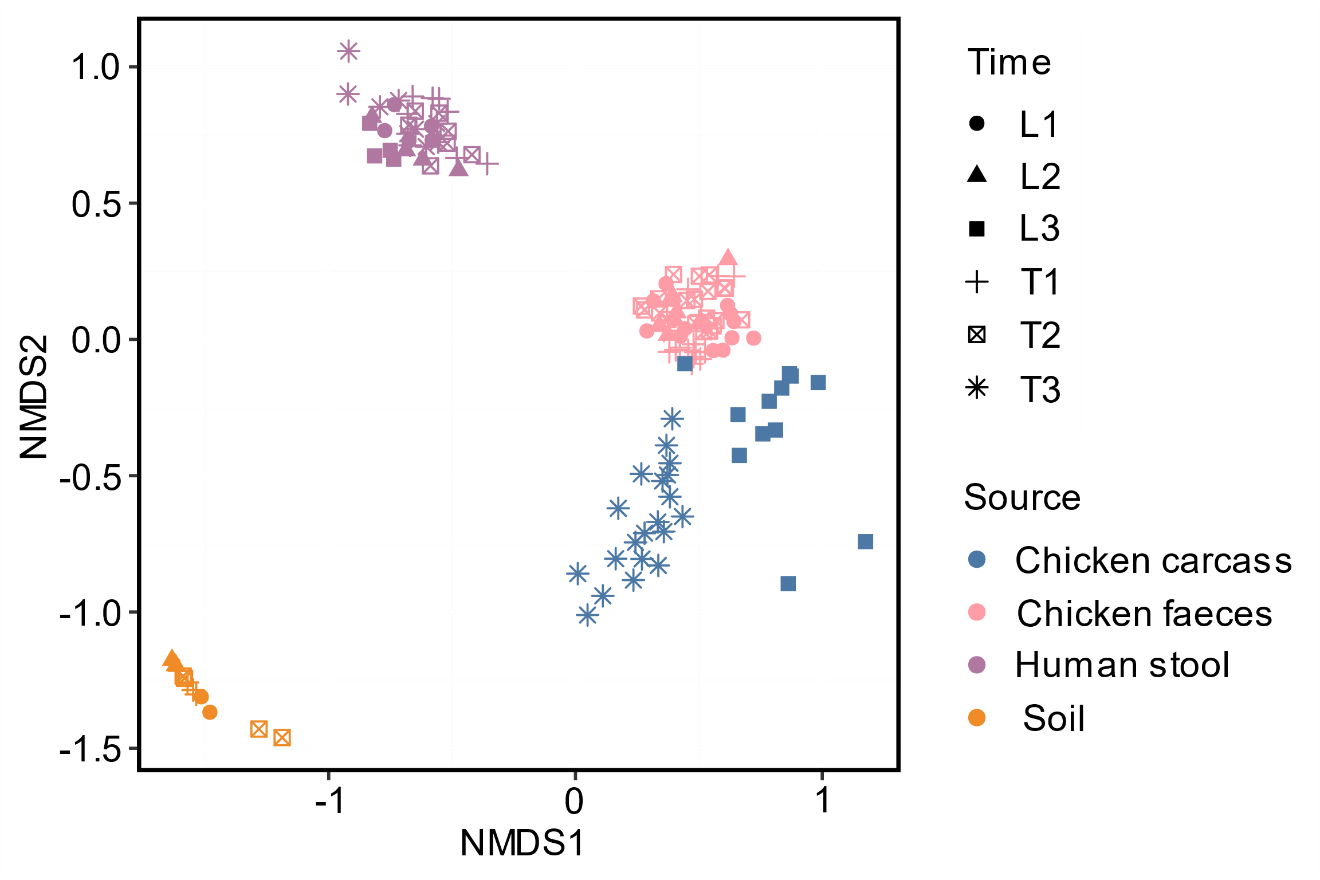
**

**Figure S4.** **NMDS analysis of species abundance among sources and timepoints**. NMDS analysis of the relative abundance of microbiome species in samples with broiler carcasses (blue), broiler faeces (pink), human faeces (purple) and soil (orange), based on Bray-Curtis dissimilarity.

**
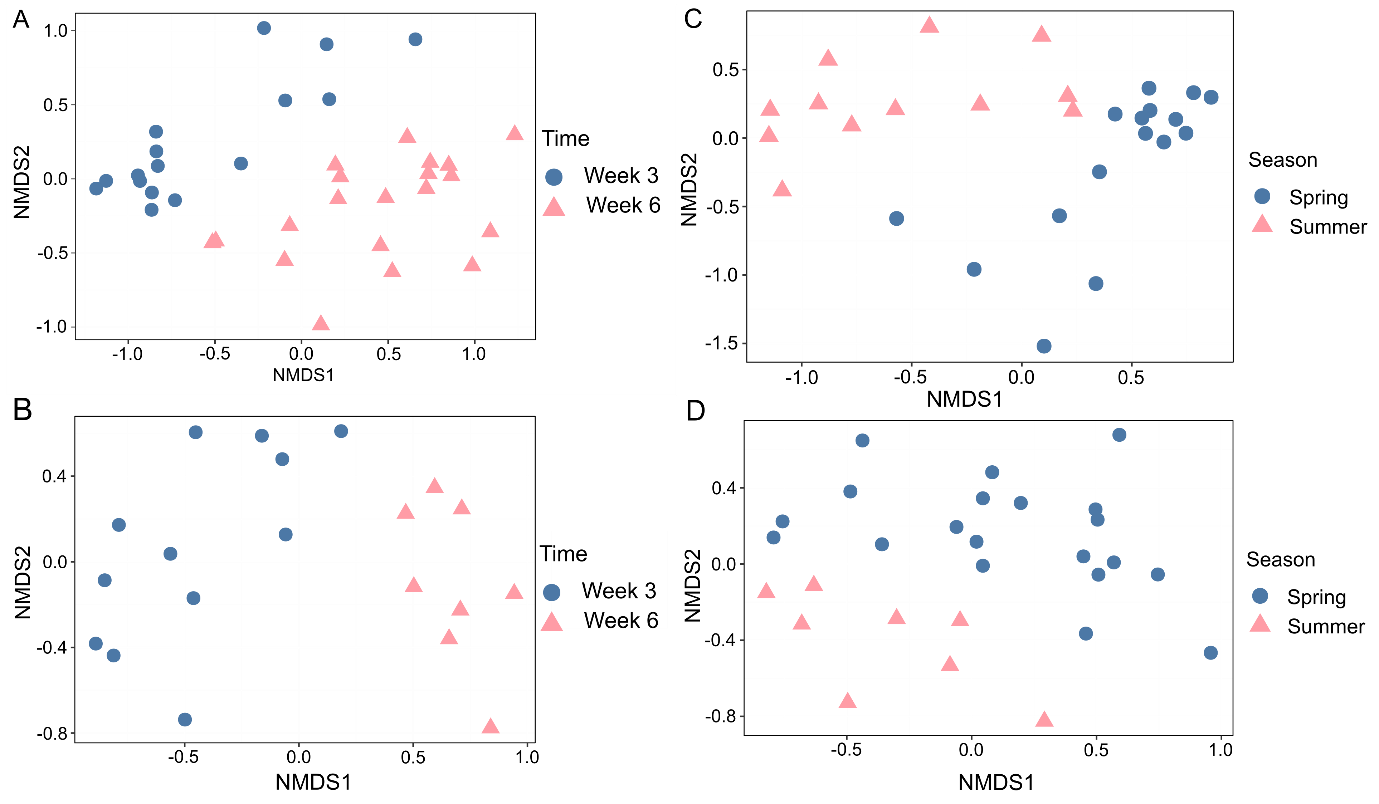
**

**Figure S5.** **NMDS analysis of species abundance among different growth time and season collection**. **(A)** Spring collection broiler faeces clustered by sample collection time (PERMANOVA: P < 0.001). **(B)** Summer collection broiler faeces clustered by sample collection time (PERMANOVA: P < 0.001). **(C)** Broiler faeces samples taken at T1 and L1 (week 3) clustered by season collection (PERMANOVA: P < 0.001). **(D)** Broiler faeces samples taken at T2 and L2 (week 6) clustered by season collection (PERMANOVA: P < 0.001).


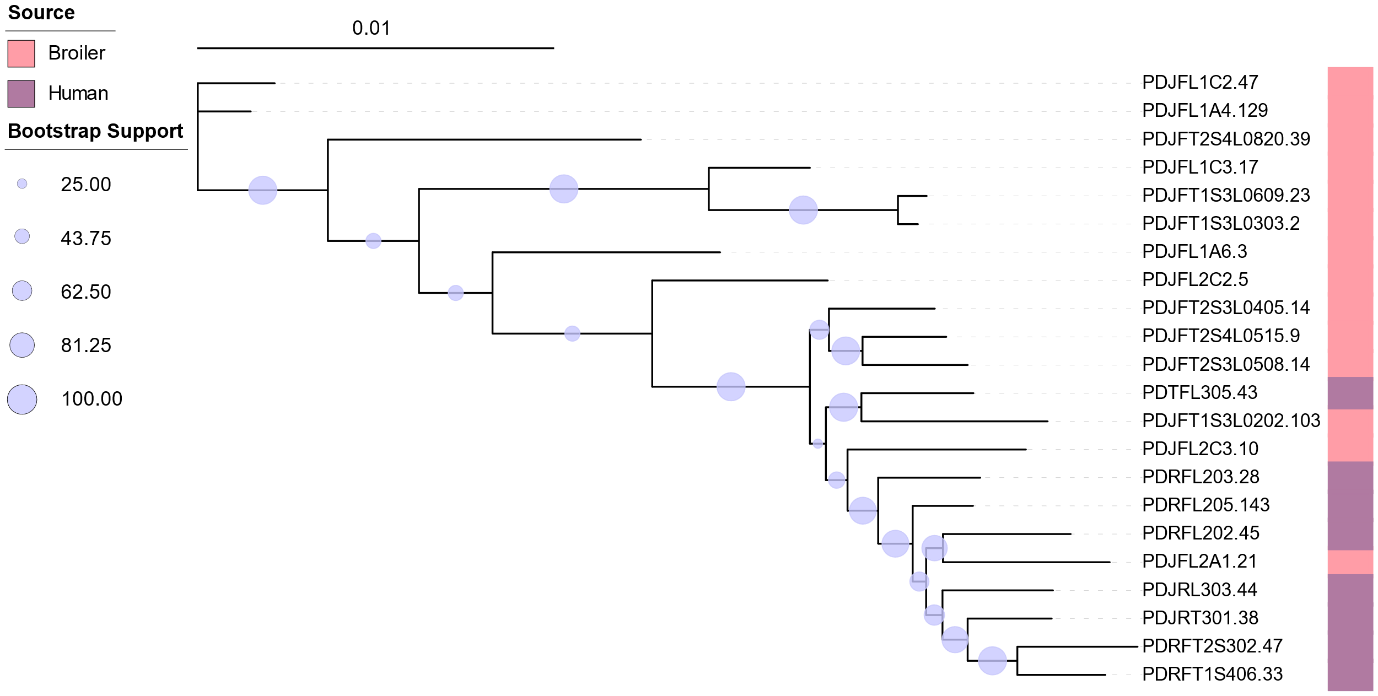


**Figure S6. Maximum likelihood phylogenetic reconstruction of 12 *E. coli* MAGs.** MAGs were assembled from human and broiler metagenome samples and a core genome alignment was used to reconstruct the phylogeny. Bootstrap support is indicated by the size of the coloured circles. The sample host is indicated by the coloured strip with human (purple) or broiler (pink) indicated.

**
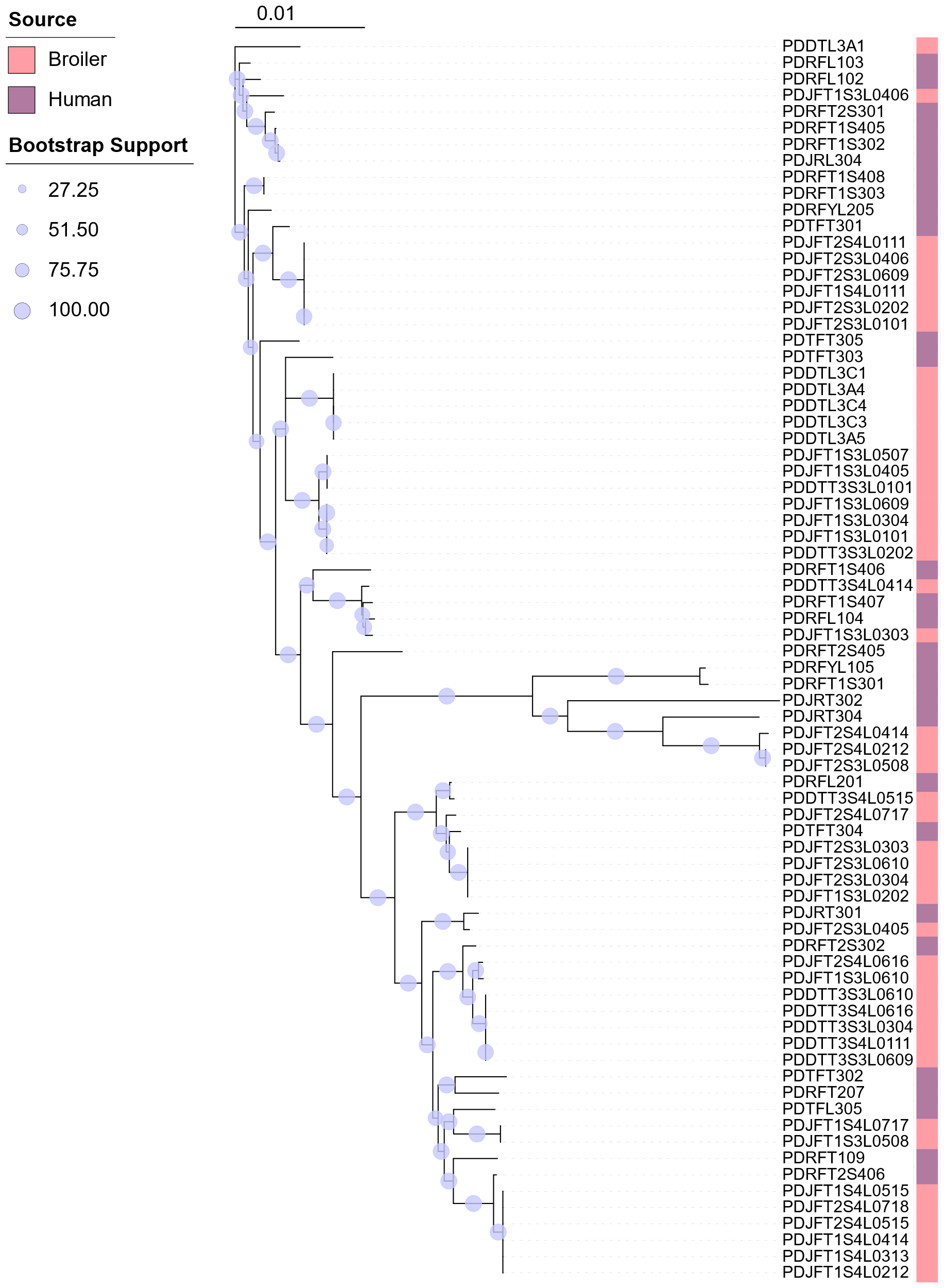
**

**Figure S7. Maximum likelihood phylogenetic reconstruction of 76 *E. coli* isolates.** Whole genome sequences of *E. coli* strains isolated from cultures taken from 76 of the human and broiler samples (30 human and 46 chicken) for which we had also metagenomic sequences were assembled (13). A core genome alignment was used to reconstruct the phylogeny. Bootstrap support is indicated by the size of the coloured circles. The sample host is indicated by the coloured strip with human (purple) or broiler (pink) indicated.

**
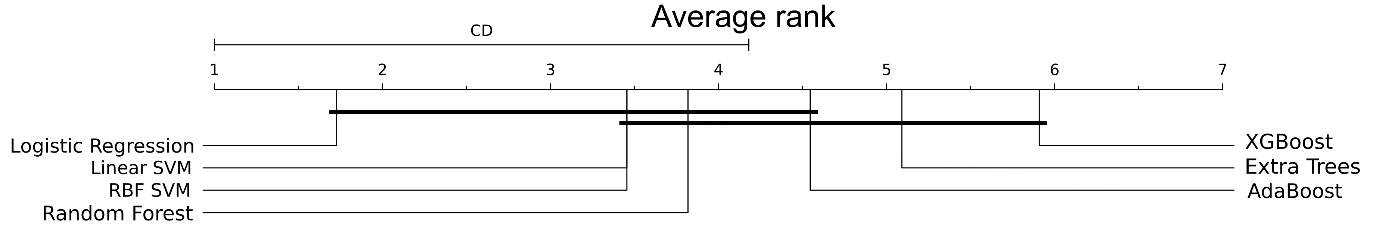
**

**Figure S8. Nemenyi *post-hoc* test.** Comparison of the performance of the 5 classifiers and 2 meta-methods, using their average ordinal rank over the 11 antibiotics analysed based on the AUC performance metric. The x-axis indicates the average ordinal rank of the machine learning methods. The scale is from 1 (best rank) to 7 (worst rank). The ordinal rank of a classifier is defined as follows: the ML method with the best AUC is given rank 1, the second-best AUC rank 2 and the *n*-th AUC best rank *n*, with *n* being the number of machine learning methods used. For each antibiotic, the methods are ranked between 1 (highest AUC) and 7 (lowest AUC), since in this case there are 7 machine learning methods used. Next, for each method, the ranks are averaged based on the 11 antibiotics studied. Logistic regression has the best average rank, which indicates that this is the method with the best average AUC performance for correlating ARGs (features) present in the broiler chicken faecal metagenomes and the antimicrobial resistance/susceptibility profiles of cultured *E. coli* from the same sources. The critical distance (CD) is defined based on the Nemenyi *post-hoc* test, all the methods that fall in the same bold bar below the axis are considered statistically equivalent based on the CD value.


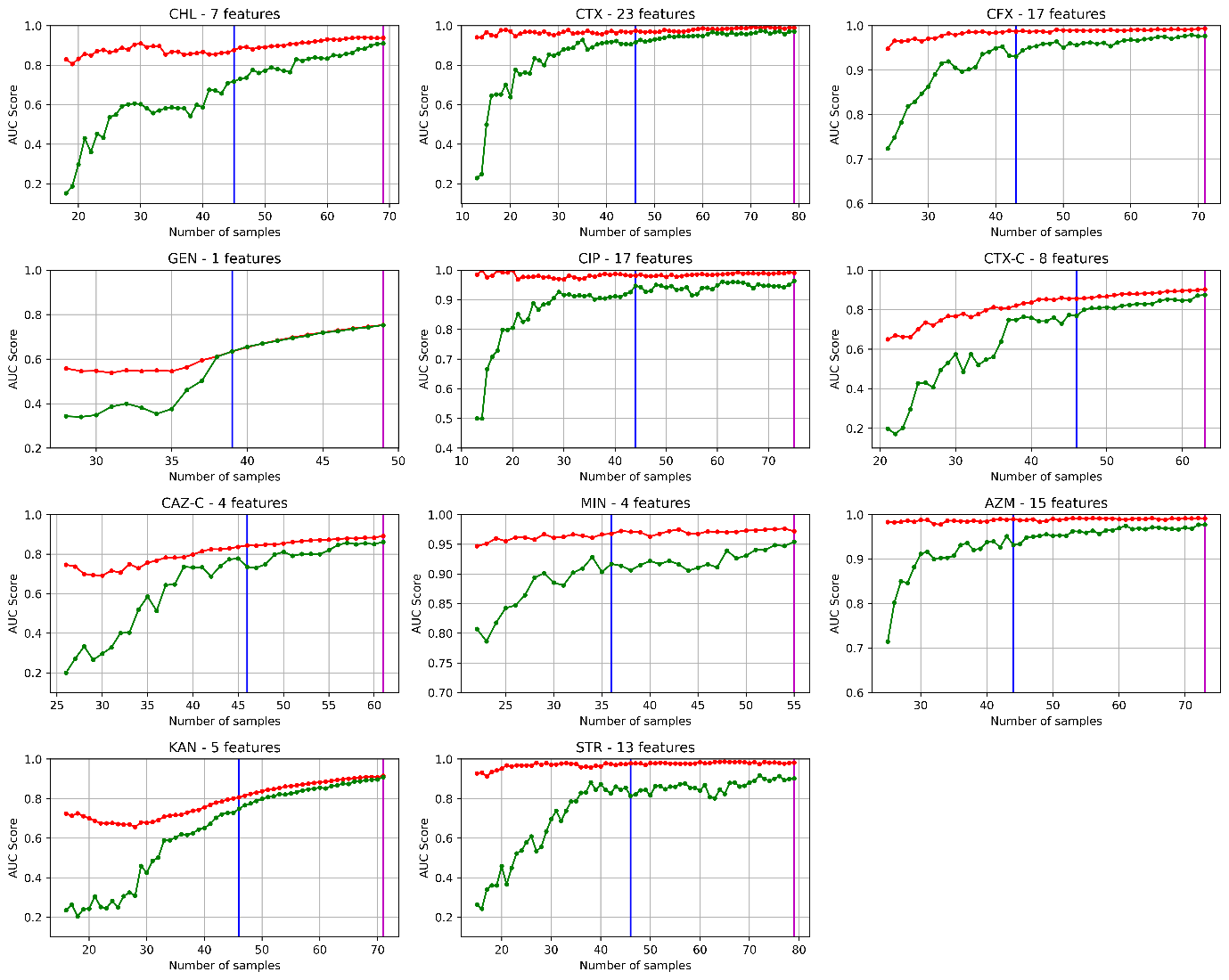


**Figure S9. Learning curves of the 11 antibiotic models trained on broiler chicken resistome data.** For each antibiotic, a wrapper backward selection approach was used to evaluate the training and testing performance whilst decreasing the number of samples. The red line indicates the accuracy of the training set whilst the green line indicates the accuracy of the test set. The vertical blue line is indicative of the original number of samples and the vertical purple line shows the number of samples after SMOTE was applied to balance the data.


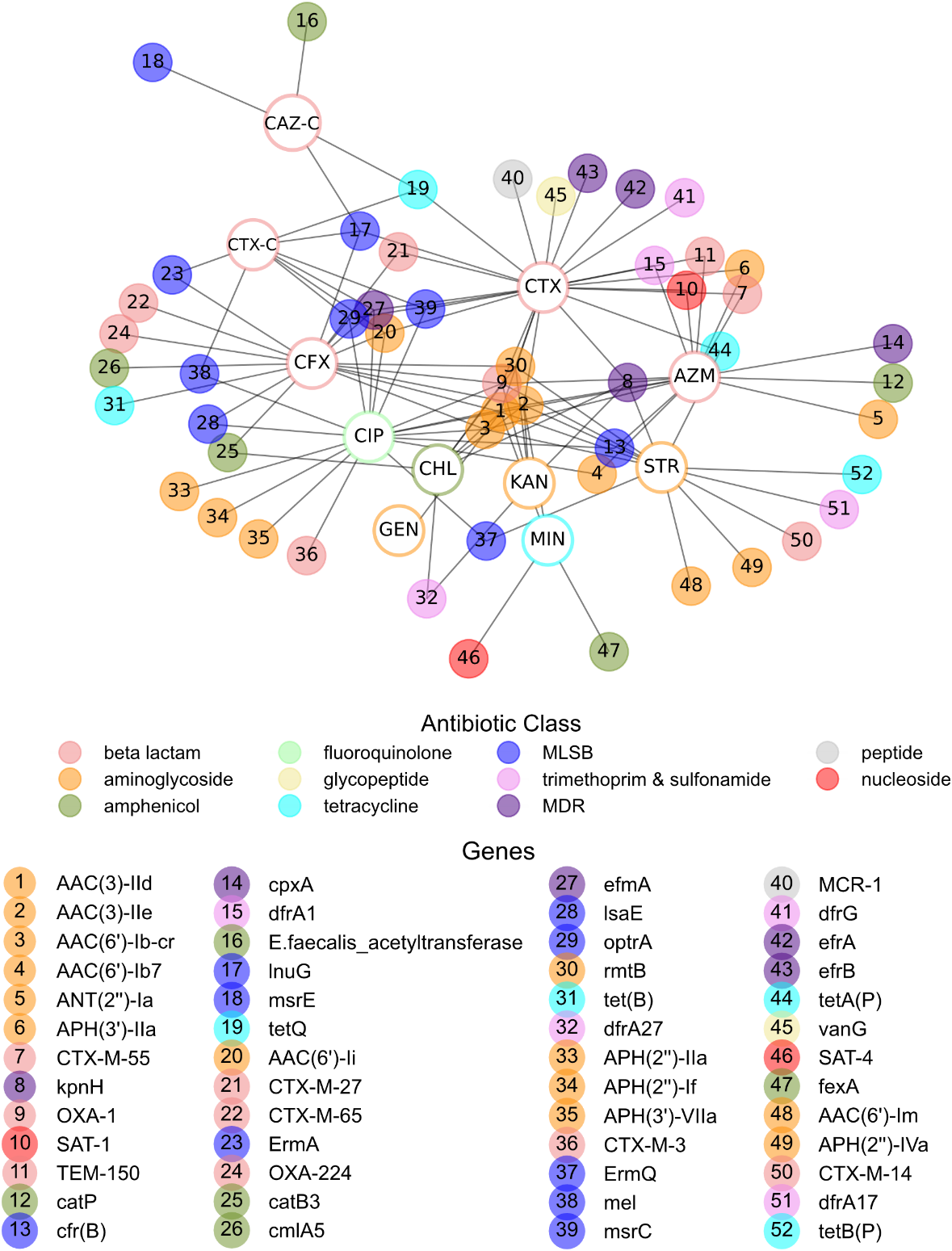


**Figure S10. Undirected graph of the ARGs in the broiler resistome found associated with the antimicrobial resistance/susceptibility profiles of *E coli* isolates collected from the same samples using supervised machine learning**. Edges of the graph link the ARG nodes (predictor variables) to the antibiotic model they were found to be predictive in. Both the ARG and antibiotic nodes are colour coded according to antibiotic class. Multidrug resistance genes are colour coded separately. The machine learning models were run for the following antibiotics: aztreonam (AZM), cefotaxime (CTX), cefotaxime/clavulanic acid (CTX-C), cefoxitin (CFX), ceftazidime/clavulanic acid (CAZ-C), chloramphenicol (CHL), ciprofloxacin (CIP), gentamicin (GEN), kanamycin (KAN), minocycline (MIN), streptomycin (STR).


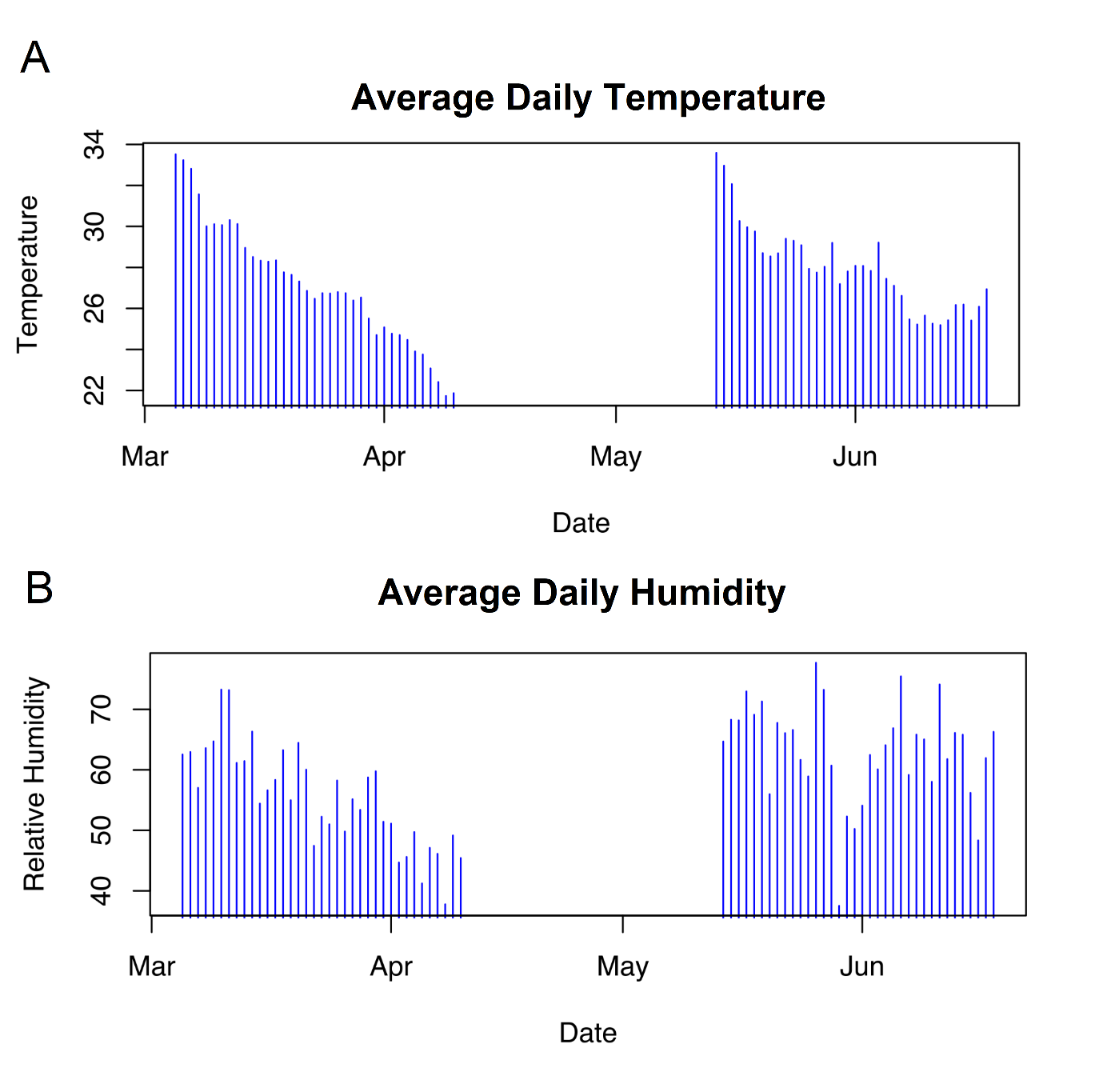


**Figure S11. Bar chart of the humidity and temperature recorded over time for the sampled broiler farm. (A)** Average daily temperature recorded in spring and summer. **(B)** Average daily humidity recorded in spring and summer.

**
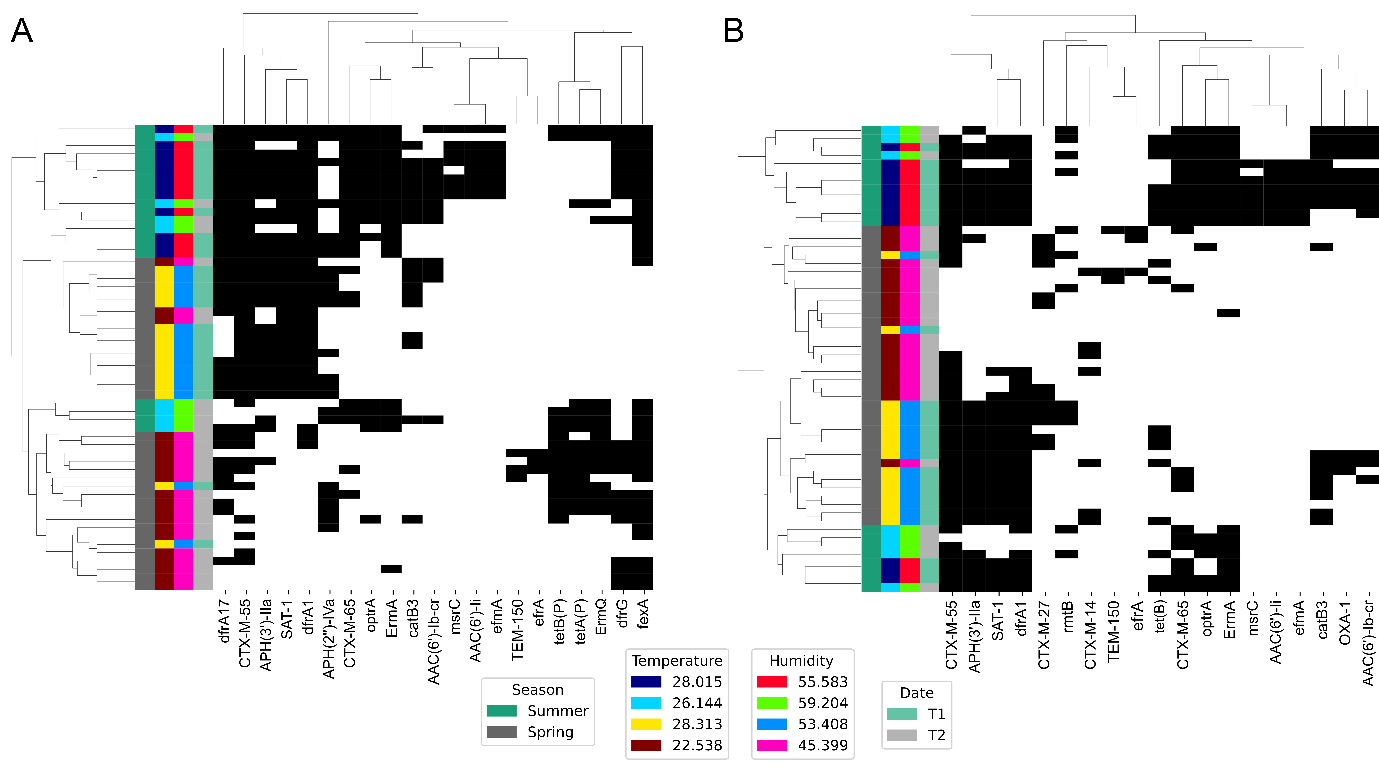
**

**Figure S12. Hierarchically clustered heatmaps of the presence (black) or absence (white) of ARGs used in regression analysis (A)** Heatmap of the presence-absence of genes used in the regression of the temperature **(B)** Heatmap of the presence-absence of genes used in the regression of the temperature

**List of Supplementary Tables**

**Supplementary Table 1.** Sample Collection Information. This includes sample ID, sequence accession data, read counts, production cycle, sample collection date, sample source, sample collection place (Farm/Abattoir) and *E. coli* AMR testing results for the samples.

**Supplementary Table 2.** Construction of the broiler faeces and carcass MAGs, including GTDB-tk assignment, ANI value with the reference accession ID, species representative, and genome assessment information including completeness, contamination level and N50.

**Supplementary Table 3.** Construction of the human faeces MAGs, including GTDB-tk assignment, ANI value with the reference accession ID, and genome assessment information.

**Supplementary Table 4.** Construction of the soil MAGs, including GTDB-tk assignment, ANI value with the reference accession ID, and genome assessment information.

**Supplementary Table 5.** Taxonomic profiling of the metagenomic samples, including the sample source, collection time and MetaPhlAn 3.0 (14) assignment.

**Supplementary Table 6.** ARG types identified in this study, including the resistance gene accession IDs in the CARD database, gene names, antibiotic drug class assignment and resistance mechanisms.

**Supplementary Table 7**. Characteristics of antibiotics resistance genes in the collected samples. For each gene a description of the sample ID, sample source and collection time is given. The occurrence (presence/absence) of each gene in each sample source at each time point is assigned as a binary code of 0 and 1 indicating absence or presence, respectively.

**Supplementary Table 8**. Shared mobile ARGs of clinically relevant antimicrobial resistance genes (ARGs) and associated mobile genetic elements found within broiler and human samples. Contigs were classed as mobile ARGs where the MGE was in close vicinity (5kb) of the ARG. For each clinically relevant gene analysed, the name and type of MGEs have been provided together with the host type, the number of samples where the genes with associated MGEs were found and the distance between the ARG and MGE (in bp).

**Supplementary Table 9.** Prediction performance results of the supervised machine learning classification for the correlation of the metagenome shotgun sequencing data (ARG presence-absence) and *E. coli* resistance/susceptibility profiles (isolates cultured from the same samples) against a panel of antimicrobials. The prediction metrics used were calculated as accuracy (TP+TN/(P+N)), sensitivity (true positive rate: TP/P), specificity (true negative rate: TN/N), AUC and precision. The scores for each performance metric were computed from 30 simulations using nested cross-validation. The mean ± standard deviation of these 30 iterations was then used as the result statistics for the performance.

**Supplementary Table 10.** Humidity and temperature recordings during spring and summer production cycles for the sampled broiler farm.

**Supplementary Table 11.** Genes significantly associated with temperature or humidity based on regression analysis, using a Wald Test with t-distribution of the test statistic. Genes to test were selected based on them having a significant association with AMR profiles of cultured *E. coli* isolates.

**Supplementary Table 12** Antibiotics given to broilers in each production cycle over their lifetime.

**Supplementary Table 13** MAGs shared between human and broiler faeces.

**References**

1. Glendinning L, Stewart RD, Pallen MJ, Watson KA, Watson M. Assembly of hundreds of novel bacterial genomes from the chicken caecum. Genome Biol. 2020;21(1):1-16.

2. Stewart RD, Auffret MD, Warr A, Wiser AH, Press MO, Langford KW, et al. Assembly of 913 microbial genomes from metagenomic sequencing of the cow rumen. Nat Commun. 2018;9(1):870.

3. Li D, Liu CM, Luo R, Sadakane K, Lam TW. MEGAHIT: an ultra-fast single-node solution for large and complex metagenomics assembly via succinct de Bruijn graph. Bioinformatics. 2015;31(10):1674-6.

4. Li H, Durbin R. Fast and accurate short read alignment with Burrows-Wheeler transform. Bioinformatics. 2009;25(14):1754-60.

5. Parks DH, Imelfort M, Skennerton CT, Hugenholtz P, Tyson GW. CheckM: assessing the quality of microbial genomes recovered from isolates, single cells, and metagenomes. Genome Res. 2015;25(7):1043-55.

6. Asnicar F, Weingart G, Tickle TL, Huttenhower C, Segata N. Compact graphical representation of phylogenetic data and metadata with GraPhlAn. PeerJ. 2015;3:e1029.

7. Chaumeil PA, Mussig AJ, Hugenholtz P, Parks DH. GTDB-Tk: a toolkit to classify genomes with the Genome Taxonomy Database. Bioinformatics. 2019;36(6):1925-7.

8. Medvecky M, Cejkova D, Polansky O, Karasova D, Kubasova T, Cizek A, et al. Whole genome sequencing and function prediction of 133 gut anaerobes isolated from chicken caecum in pure cultures. BMC Genomics. 2018;19(1):561.

9. Diaz Carrasco JM, Casanova NA, Fernández Miyakawa ME. Microbiota, Gut Health and Chicken Productivity: What Is the Connection? Microorganisms. 2019;7(10).

10. Iman RL, Davenport JM. Approximations of the critical region of the fbietkan statistic. Communications in Statistics - Theory and Methods. 1980;9(6):571-95.

11. Demšar J. Statistical comparisons of classifiers over multiple data sets. The Journal of Machine Learning Research. 2006;7:1-30.

12. Chawla NV, Bowyer KW, Hall LO, Kegelmeyer WP. SMOTE: synthetic minority over-sampling technique. Journal of artificial intelligence research. 2002;16:321-57.

13. Peng Z, Maciel-Guerra A, Baker M, Zhang X, Hu Y, Wang W, et al. Whole-genome sequencing and gene sharing network analysis powered by machine learning identifies antibiotic resistance sharing between animals, humans and environment in livestock farming. PLoS Comput Biol. 2022;18(3):e1010018.

14. Segata N, Waldron L, Ballarini A, Narasimhan V, Jousson O, Huttenhower C. Metagenomic microbial community profiling using unique clade-specific marker genes. Nat Methods. 2012;9(8):811-4.
